# Conservative Spruce Outperforms Acquisitive Larch Under Warming and CO_2_

**DOI:** 10.1111/gcb.71157

**Published:** 2026-10-24

**Authors:** Yanjun Song, Alexandria Pivovaroff, Mahmuda Islam, Martin P. Girardin, Alana Chin, Hervé Cochard, José M. Torres‐Ruiz, José A. Medina‐Vega, Sylvain Delzon, Xiaohan Yin, Xiao Jing Guo, Mirindi E. Dusenge, Jeffrey M. Warren, Paul J. Hanson, Peter B. Reich, Andrew D. Richardson, Lingcheng Li, Avni Malhotra, Verity G. Salmon, Nate G. McDowell

**Affiliations:** ^1^ School of Biological Sciences Washington State University Pullman Washington USA; ^2^ Biology Department Occidental College Los Angeles California USA; ^3^ Department of Forestry & Environmental Science Shahjalal University of Science and Technology Sylhet Bangladesh; ^4^ Natural Resources Canada Canadian Forest Service, Laurentian Forestry Centre Quebec City Quebec Canada; ^5^ Department of Biological Sciences Cal Poly Humboldt Arcata California USA; ^6^ Université Clermont Auvergne, INRAE, PIAF Clermont‐Ferrand France; ^7^ Instituto de Recursos Naturales y Agrobiología de Sevilla (IRNAS), Consejo Superior de Investigaciones Cientıficas (CSIC) Seville Spain; ^8^ Forest Global Earth Observatory Smithsonian Tropical Research Institute Washington DC USA; ^9^ INRAE, Univ. Bordeaux, UMR BIOGECO Pessac France; ^10^ Department of Medical Biochemistry and Biophysics Umeå universitet Umeå Sweden; ^11^ ANU Research School of Biology The Australian National University Canberra Australia; ^12^ Climate Change Science Institute and Environmental Sciences Division Oak Ridge National Laboratory Oak Ridge Tennessee USA; ^13^ Institute for Global Change Biology, and School for Environment and Sustainability University of Michigan Ann Arbor Michigan USA; ^14^ Department of Forest Resources University of Minnesota St. Paul Minnesota USA; ^15^ Hawkesbury Institute for the Environment Western Sydney University Penrith Australia; ^16^ Center for Ecosystem Science and Society Northern Arizona University Flagstaff Arizona USA; ^17^ Of Informatics, Computing, and Cyber Systems Northern Arizona University Flagstaff Arizona USA; ^18^ Atmospheric Sciences and Global Change Division Pacific Northwest National Lab Richland Washington USA; ^19^ Biological Sciences Division Pacific Northwest National Laboratory Richland Washington USA

**Keywords:** black spruce, bog, carbon assimilation and hydraulics, functional strategies, nutrients, tamarack, whole‐ecosystem warming

## Abstract

Predicting boreal forest growth is hindered by limited experimental tests on the compounding effects of climate change and atmospheric CO_2_. We integrated regional tree‐ring data with a peatland warming and CO_2_ manipulation experiment, including 45 trait measurements, to assess growth responses of two widespread boreal conifers, *Larix laricina* and *Picea mariana* , to temperature, vapor pressure deficit (VPD), soil moisture, and elevated CO_2_. *L. laricina* growth was consistently more climate‐sensitive than *P. mariana* , increasing strongly with soil moisture and declining slightly with VPD at the regional scale, but responding positively to VPD and temperature—not soil moisture or CO_2_—at the experimental peatland. *L. laricina* 's acquisitive strategy enhanced water and nutrient uptake, sustaining growth but reducing hydraulic safety. By contrast, *P. mariana* 's conservative strategy maintained water‐use efficiency and hydraulic resilience without compromising growth. Our results challenge projections of widespread CO_2_ fertilization in boreal forests and highlight conservative conifers as potential winners under warming and drying, sustaining growth while maintaining low mortality risk. This dynamic could reshape projections of the boreal carbon sink and its role in global climate feedback.

## Graphical abstract

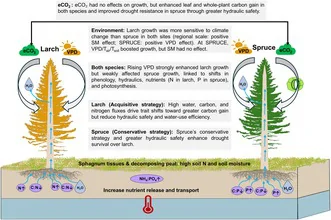

## 1 Introduction

Boreal forests constitute ~30% of global forests and are pivotal to carbon storage and climate regulation (Gauthier et al. 2015). However, rapid environmental changes (Scheffer et al. 2012) are driving divergent tree growth and mortality across species and regions (Chen et al. 2023; Reich et al. 2022; Sterck et al. 2024; Walker et al. 2015; Wang et al. 2023), creating uncertainty for forest carbon dynamics. Boreal tree growth is influenced by the interacting drivers of temperature, elevated CO_2_ (eCO_2_), vapor pressure deficit (VPD), soil moisture (SM), and nutrient availability. Warming and eCO_2_ promote growth via longer growing seasons (Reyes‐Fox et al. 2014; Richardson et al. 2018) and nutrient availability (Gao et al. 2022; Jarvis and Linder 2000; Norby et al. 2024) and increased water‐use efficiency (McDowell et al. 2022). Conversely, rising VPD exacerbates hydraulic failure risk and reduces growth via stomatal closure and suppressed photosynthesis (Dusenge et al. 2019; Grossiord et al. 2020; López et al. 2021; McDowell et al. 2016; Mirabel et al. 2023). In wet environments like peatlands, however, ample soil water buffers evaporative demand, and higher temperatures can even enhance photosynthesis (Chen et al. 2023; Dusenge et al. 2023). Soil warming may further promote growth via increased hydraulic lift (Wieser et al. 2015) and nutrient availability (Iversen et al. 2023; Jarvis and Linder 2000; Malhotra et al. 2020). Understanding these interacting drivers is critical for predicting boreal forest response to climate change.

Tree functional strategies for carbon, water, and nutrient acquisition and allocation, particularly along the acquisitive‐conservative continuum, from enzyme to whole‐plant scales, probably mediate divergent growth responses to warming and eCO_2_ (Díaz et al. 2016; Reich et al. 2014; Tilman 1988). Acquisitive species maximize photosynthesis and resource uptake to achieve high growth, but limited investment in survival traits (e.g., embolism resistance) increases mortality risk (Torres‐Ruiz et al. 2024). In contrast, conservative species under rising VPD may benefit less from eCO_2_ because greater stomatal closure increases water‐use efficiency and maintains hydraulic safety but limits photosynthesis (Song et al. 2022).

These strategies reflect species' positions along the resource economics spectrum (RES) (Reich 2014) through trade‐offs among hydraulics, photosynthesis, and nutrient acquisition. Traits that increase water transport often reduce hydraulic safety, with trade‐offs driven by stomatal conductance, leaf and xylem anatomy, embolism vulnerability, and leaf area (Sperry et al. 1998; Torres‐Ruiz et al. 2024). Photosynthesis under eCO_2_ can be enhanced by nutrient allocation to greater leaf area and photosynthetic capacity (Dusenge et al. 2021), but at the expense of hydraulic safety (Ewers et al. 2000; McDowell et al. 2022). Trees must balance such trade‐offs by reallocating nutrients among leaves, roots, and wood to optimize growth as soil warming increases nitrogen and phosphorus availability (Ellsworth et al. 2022; Iversen et al. 2023, 2008), which influence their ability to overcome nutrient constraints under eCO_2_ (Jiang et al. 2020; Wang et al. 2020). Yet despite evidence that conservative species sustain growth under stress while acquisitive species falter (Augusto et al. 2025), most terrestrial biosphere models (TBM) still lack mechanistic representations of how multiple drivers shape these contrasting strategies (Ellsworth et al. 2022; Kattge et al. 2009).

Trait shifts could further influence tree growth and survival under climate stress and rising CO_2_. We define trait shifts, or acclimation, as longer‐term, reversible adjustments in anatomy, structure, or biochemistry in response to environmental drivers (Fonti et al. 2013; Gunderson et al. 2010; Sterck et al. 2016). Hydraulic, photosynthetic, and nutrient acquisition traits can shift to maximize resource capture or survival for gas exchange acclimation (Charrier et al. 2018; Gessler et al. 2017; Limousin et al. 2013; Sperry et al. 2019). Boreal trees have exhibited photosynthetic shifts under eCO_2_ (Dusenge et al. 2024), temperature (Dusenge et al. 2023), and soil moisture (Reich et al. 2018). Despite the key role trait shifts could play in future boreal forest dynamics, TBMs rarely capture these shifts due to limited experimental evidence from mature trees under compounding drivers (De Kauwe et al. 2017).

The scarcity of experimental tests hampers mechanistic insight into compounding drivers, limiting predictions of boreal forest responses to future conditions. *Larix laricina* (larch; more acquisitive) and *Picea mariana* (black spruce; more conservative) dominate and co‐occur in high‐latitude boreal forests and are pivotal to global carbon and water cycling. Here, we investigated their growth responses and underlying mechanisms to rising temperature (T_air_ and T_soil_), VPD, SM, and eCO_2_. We hypothesize that: (1) high VPD would be negatively correlated with growth due to stomatal closure and increased embolism, while increased water‐use efficiency (Walker et al. 2015) from eCO_2_ would offset these negative correlations; (2) rising T_soil_ and SM would enhance growth via greater nutrient availability and lower hydraulic risk; (3) larch, with its acquisitive strategy, would exhibit greater resource capture and growth under warming and eCO_2_ than spruce, but at the cost of hydraulic risk from rising VPD; and (4) larch's acquisitive strategy would enable greater acclimation to changing environmental drivers than spruce due to its greater resource capture and hence ability to shift resource allocation.

To test our four hypotheses, we integrated regional‐scale dendrochronology from 115 Canadian sites (1970–2010, Figure 1a) with an experimental peatland warming and eCO_2_ manipulation (Spruce and Peatland Responses Under Changing Environments; SPRUCE, 2017–2021) (Warren et al. 2021), where we measured 45 traits related to photosynthesis and leaf anatomy, hydraulics and wood anatomy, and nutrients (Table S1). For both species, we calculated basal area increment (BAI; cm^2^ year^−1^) at both scales and used generalized linear mixed models to investigate long‐term growth–climate relationships. Using experimental trait data, we performed principal component analysis (PCA) to identify species‐specific strategies ranging from conservative to acquisitive based on integrated traits along the RES and to quantify trait gradients (Figure 2). In larch, the first principal component (PC1) described a whole‐tree RES, increasing with crown greenness, growing season length, transpiration and nitrogen, and decreasing with water potential (Figure 2b). In spruce, PC2 described a leaf‐level RES, increasing with photosynthesis, transpiration and water‐use efficiency, and decreasing with anisohydric tendency (Figure 2c). We used PC1 and PC2 to evaluate growth strategies under rising VPD. We focused on VPD as a physiological stressor because it directly regulates transpiration demand and hydraulic function, whereas T_air_ influences growth through photosynthesis, respiration, and phenology (Reich et al. 2022) without direct links to xylem and stomatal function. By integrating temperature, VPD captures key constraints on plant water use under climate change (McDowell et al. 2022). To reveal trait mechanisms underlying growth responses to rising VPD, we regressed BAI or VPD against traits and soil nutrients. Given limited sample size under CO_2_ treatments (*n* = 4–5 per species), we assessed significance using an exact two‐sided permutation test (Methods).

**FIGURE 1:**
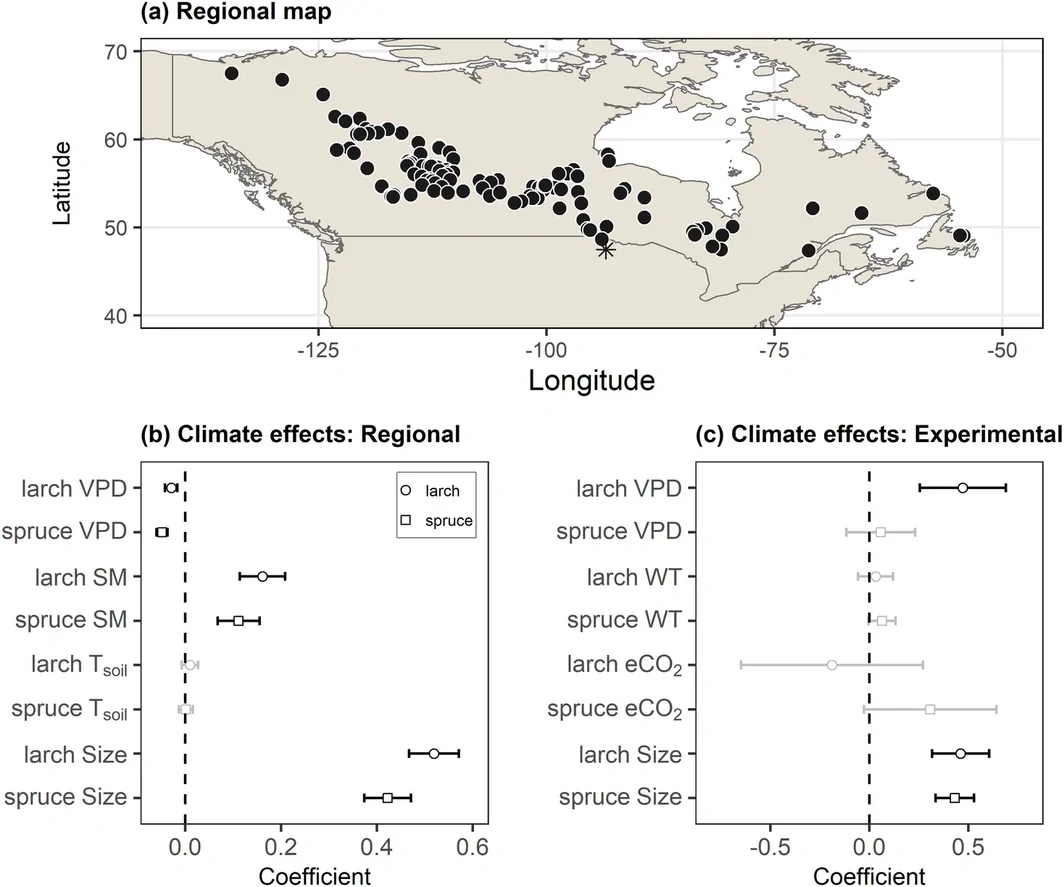
Effects of vapor pressure deficit (VPD), soil temperature (T_soil_), and soil moisture (SM) or soil water table depth from surface (WT) on basal area growth for larch (i.e., *L. laricina* ) and spruce (i.e., *P. mariana* ) across Canada and at the SPRUCE Ecosystem experiment. (a) A regional distribution map in Canada across 115 sites. The asterisk (*) denotes the SPRUCE experimental site. Map lines delineate study areas and do not necessarily depict accepted national boundaries. (b) Growth‐climate relationships at the regional scale for VPD, T_soil_, and SM from 1970 to 2010. (c) Growth‐climate relationships at the SPRUCE experimental scale for VPD, T_soil_, and WT from 2017 to 2020. The x axes indicate the values of standardized coefficients with error bars (95% confidence intervals), and the y axes indicate climate and tree size factors (b, c). Grey symbols indicate that the standardized coefficients (b, c) are not different from zero.

**FIGURE 2:**
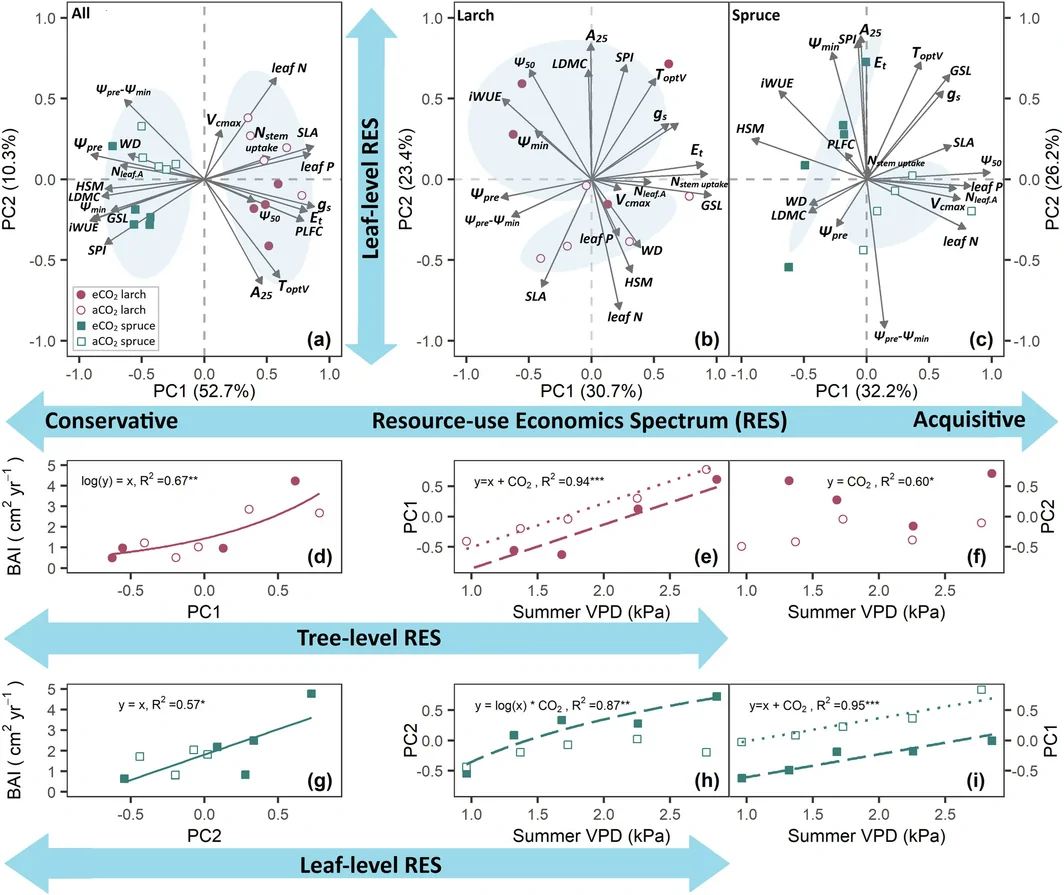
Principal components analysis (PCA) of functional traits shows a resource economics spectrum (RES) at the experimental site. (a–f) The first two PCA axes and the loadings of 21 traits are shown. PC1 (x‐axis) and PC2 (y‐axis) indicate the tree‐level and leaf‐level RES, respectively. (d–f) Relationships between tree‐level RES, basal area increment (BAI), and summer vapor pressure deficit (VPD), and a more acquisitive leaf‐level strategy under elevated CO_2_ (eCO_2_) in larch. (g–i) Relationships between leaf‐level RES, BAI, and summer VPD, and a shift toward a more acquisitive tree‐level strategy with increasing VPD in spruce. Red and teal indicate larch and spruce, respectively. Filled and open symbols indicate elevated (eCO_2_) and ambient CO_2_ (aCO_2_), respectively. Solid, long‐dashed, and dotted lines indicate significant regressions across CO_2_ treatments, under aCO_2_, and under eCO_2_, respectively. For trait abbreviations, see Table S1.

## 2 Methods

### 2.1 Study Sites and Experiment Design

#### 2.1.1 Regional‐Scale Tree Ring and Climate Datasets

To assess climate impacts on stem area growth for *Picea mariana* (Mill.) B.S.P. (spruce) and *Larix laricina* (Du Roi) K. Kochlarch (larch or tamarack) regionally, we obtained tree‐ring datasets from 1951 to2017 in 115 sites across Canadian boreal areas (Figure 1a) from CFS‐TRenDv1.1 (Girardin et al. 2021). To exclude the disturbance from the sawfly outbreak in the 1960s and low sample size, we used the tree‐ring data from 1970 to 2010 for further analyses. To ensure comparability of growth responses, we filtered the dataset to include only sites where both spruce and larch coexisted. Sites where spruce and larch coexist are typically nutrient‐poor conditions, characterized by moist, organic‐rich soils, often found in lowland boreal environments such as peatlands, forested wetlands, and poorly drained depressions. Annual basal area increment (BAI, cm^2^ year^−1^) was calculated from annual tree ring‐width measurements to indicate stem area growth (Girardin et al. 2024; Mirabel et al. 2023). Climate data from 1970 to 2010 were extracted based on site coordinates. Mean annual temperature (MAT, °C), annual precipitation (MAP, mm), and daily VPD were obtained from Mirabel et al. (2023) using the BioSIM software with the resolution of 1° × 1° grid (Régnière et al. 2014). MAT and MAP were averaged by site across all years. These areas cover a long transect across a temperature gradient ranging from the warmest southern border (MAT > 5°C) to the coldest northern border (MAT < −5°C), and a precipitation gradient of the west coast (MAP > 1500 mm) and northern regions (MAP < 400 mm) (Girardin et al. 2024). Monthly soil moisture and temperature at 0–30 cm were extracted from ERA5‐Land data (the land component of the fifth generation of European ReAnalysis (Muñoz‐Sabater et al. 2021)) using Google Earth Engine with a resolution of 0.1° × 0.1° grid (Gorelick et al. 2017). VPD, soil moisture (SM), soil temperature (T_soil_), and air temperature (T_air_) were averaged based on the summer season (June–August) per site per year and were used for further analyses.

#### 2.1.2 Whole Ecosystem Warming Experiment and Elevated CO_2_ Experiment

The experiment is located at the U.S. Forest Service's Marcell Experimental Forest, in Minnesota, USA (47°30.476′ N; 93°27.162′ W), the Oak Ridge National Laboratory's SPRUCE (Spruce and Peatland Responses Under Changing Environments). For detailed information about experiment design, see Hanson et al. (2017). MAT and MAP are 4°C and 750 mm, respectively (Richardson et al. 2018). This boreal peatland forest is around 50 years old at the southern border (Sebestyen et al. 2011). The dominant canopy species are *P. mariana* and *L. laricina* , and dominant understory species are ericaceous shrubs *Rhododendron groenlandicum* and *Chamaedaphne calyculata* (Dusenge et al. 2023). Ten large open‐top octagonal enclosures, each 12.8 m in diameter, 7 m in height, and 114.8 m^2^ in area, were initiated with five warming treatments (+0°C, +2.25°C, +4.5°C, +6.75°C, and +9°C) in August 2015. Elevated CO_2_ (eCO_2_, +500 ppm) treatments were added to five enclosures in June 2016, and the remaining five enclosures were ambient CO_2_ (aCO_2_).

#### 2.1.3 Climate Monitoring at the Experimental Site

Environmental measurements were monitored in the 10 enclosures since 2014, including half‐hourly precipitation, relative humidity, temperature, soil moisture, soil water table depth, and CO_2_ (Hanson et al. 2016). Since warming+CO_2_ treatments began in 2016 and climate and growth data were available from 2014 to 2021 at the time of analysis, we used data from 2017 to 2021 to focus on the post‐treatment effects of warming and CO_2_. We focused on temperature‐driven VPD, which is highly correlated with temperature treatments, as evidenced by the similar results obtained when using either VPD or temperature treatments. Firstly, we calculated half‐hourly VPD from air temperature and relative humidity at 4 m height using the method by Buck (1981). Per year per plot, we averaged half hourly VPD by day to get daily VPD and then averaged daily VPD by summer season (June–August) as summer VPD. Similarly, summer soil moisture (SM, the ratio of water volume to total soil volume) from hollow sites at the depth of 10 cm and soil water table depth from surface (WT) were calculated. WT was used instead of SM due to its high correlation with SM (*r* = 0.73, *n* = 40, *p* < 0.001) and the lack of SM in 2017. Annual climate data were used to analyze growth‐climate relationships, while averaged climate data were used to analyze trait‐climate relationships since there was no variation in VPD across years.

### 2.2 Trait Measurements at the Experimental Site

#### 2.2.1 Tree Stem Area Growth Rate (BAI)

To measure annual tree basal area growth, DBH was annually measured per individual per plot since 2011, and was extracted from the SPRUCE datasets (Hanson et al. 2018) for further analysis between 2017 and 2021. To have a consistent dataset with our hydraulic, wood anatomy, and leaf anatomy measurements, we used the same individual per species per treatment as target trees for calculating tree growth and analyzing growth‐trait relationships. To increase statistical power and have more representative growth, all individual trees per species per treatment were used for analyzing growth‐climate relationships. Annual stem basal area (BA, cm^2^) was calculated from diameter at breast height (DBH) as BA = π × DBH^2^/4. We applied the two methods below to calculate stem area growth rate (BAI, cm^2^ yr.^−1^) over 2017–2021 for our target trees and all individuals. For target trees used to analyze growth‐trait relationships, mean annual BAI from 2017 to 2021 was calculated as the difference between BA at the end of 2021 and BA at the end of 2016, divided by 5 years: BAI = (BA_2021_ − BA_2016_)/5. For all individuals used for analyzing growth‐climate relationships, annual BAI was calculated in the year t as BAI_*t*_ = BA_*t*_ − BA_*t‐1*_.

#### 2.2.2 Hydraulic‐ and Photosynthetic‐Related Measurements

Considering the very low number of trees at the experimental site, we collected two 45 cm‐long branches from one tree individual per species per plot at SPRUCE on July 28, 2023 and August 10, 2023, to measure vulnerability curves. Despite the limited number of sampled individuals, hydraulic traits exhibited consistent patterns with other traits across treatments. To quantify crown health, we also visually measured the percentage of live foliated crown (PLFC) in the field for the target trees. To minimize measurement error, PLFC was visually measured by three persons from at least three locations based on leaf colors, leaf shedding and branch desiccation (Zhang et al. 2021). We shipped branch and leaf samples from the experimental site to INRAE‐PIAF research station (Clermont‐Ferrand, France) for hydraulic measurements. To minimize water loss from branch transpiration, we removed all leaves and small twigs from the main branches and wrapped branches in wet towels inside large black plastic bags, and then packed these bags in boxes for expedited air transport. We applied the standard “Cavitron” method on the 27 cm‐length branch without bark to get vulnerability curves by generating the curves between the decreasing hydraulic conductance and increasing water tension (Cochard et al. 2005). To obtain the maximum xylem‐specific hydraulic conductivity (K_s_, kg m^−1^ MPa^−1^ s^−1^), we first measured the maximum sample conductance (K_max_, kg MPa^−1^ s^−1^) under low xylem pressure (0.75 MPa), and then multiplied K_max_ by sample length (K_max1_) and finally divided K_max1_ by sapwood area. The xylem pressure inducing 50% loss of conductivity (Ψ_50_, MPa) was calculated by fitting a sigmoidal function to the vulnerability curve (Pammenter and Van der Willigen 1998). To measure wood density and wood anatomy, we kept a 5–7 cm and a 2–3 cm branch segment per tree for wood density and wood anatomy, respectively. Wood density (WD, g cm^−2^) was calculated as the ratio of dry mass to fresh volume. After measuring WD, samples were dried and then used to measure pit traits on tangential sections using the standardized method (Delzon et al. 2010; Song, Poorter, et al. 2021). Meanwhile, we used a subbranch to measure specific leaf area (SLA, cm^2^ g^−1^) and leaf dry matter content (LDMC, g g^−1^). SLA and LDMC were measured as the scanned leaf area over dry leaf mass and leaf water‐saturated fresh mass to leaf dry mass, respectively. Pit traits related to pit size (pit aperture diameter, DPA in μm; pit membrane diameter, DPM in μm; and torus diameter, DT in μm) and pit sealing (margo flexibility, MF) were measured using the scanning electron microscopy (PhenomG2 pro; FEI, the Netherlands) at the PHENOBOIS platform. MF was calculated as the relative membrane to torus size [MF = (DPM‐DT)/DPM]. Normally, large pits are expected to benefit hydraulic conductivity, and strong pit sealing capacity benefits embolism resistance. Tracheid traits include tracheid diameter (μm), tracheid density (TD, mm^−2^) and wall thickness (Tw, μm) for both earlywood and latewood. These anatomical traits were measured on the transverse section using a light microscope (Leica DM 2500) for pictures and ImageJ v.1.52a for calculations on a transverse section, covering the bark to pith with more than 200 tracheids per sample. The hydraulic (weighted) diameter was calculated as below:

$$D=\sqrt[{4}]{\frac{1}{n}\sum_{i=1}^{n}D_{i}^{4}},$$

where *D*_*i*_ is the *i*th tracheid diameter. For detailed measurements of wood anatomy, see methods by Song et al. (2026).

To quantify water flux and other drought‐tolerant‐related traits, we extracted sap flow density (E_t_, g m^−2^ s^−1^), predawn water potential (Ψ_pre_, MPa), and midday water potential (Ψ_min_, MPa) per species per treatment from a previous study (Warren et al. 2021). To quantify plant hydraulic safety, the hydraulic safety margin (HSM, MPa) was calculated as the difference between Ψ_min_ and Ψ_50_. To quantify transpiration (E_t_), we calculated mean summer sap flow density from three consecutive years from 2019 to 2021 (Warren et al. 2025) per enclosure per species per treatment, showing no annual differences.

In September 2023, we resampled fresh leaves from the same individual at the experimental site and transported them to ETH‐IBZ Plant Ecology in Switzerland for measuring stomatal anatomy, which is closely related to photosynthesis (Sack et al. 2003). On the leaf transverse section, nail polish imprints from both abaxial and adaxial surfaces of leaves were used to measure stomatal density (SD, mm^−2^), guard cell length (Guard, μm), and epidermal cell length (μm). Considering stomatal size and number, the stomatal pore index (SPI) was calculated from stomatal density and guard cell length (SPI = Guard^2^ × SD) (Sack et al. 2003). Since SD and Guard differ on abaxial and adaxial sides, SPI was summed up to get a total value for SPI (Bucher et al. 2016). These stomatal anatomical traits were measured using the standardized method by Chin et al. (2023).

To quantify photosynthesis capacity, we obtained photosynthesis data from the study by Dusenge et al. (2023) who collected, in June and August 2017, data on the temperature response curves of net photosynthetic rates as well as thermal responses of their underlying biochemical processes. For the evergreen black spruce, the data were collected on needle cohorts that developed in the growing season of 2016. Photosynthetic capacity was indicated by stomatal conductance at 25°C (g_s_, mol m^−2^ s^−2^), net photosynthesis rate at 25°C (A_25_, μmol m^−2^ s^−1^), maximum Rubisco carboxylation rate at 25°C (V_cmax_), and thermal optimum of maximum Rubisco carboxylation rate (T_optV_, °C). The intrinsic water‐use‐efficiency (iWUE, mmol mol^−1^) was calculated as the ratio of A_25_ to g_s_. To track the timing of leaf green‐up, green‐down and growing season, cameras were installed in August 2015 to calculate phenology data (Richardson et al. 2018). Phenology data were averaged from 2017 to 2021 to match the growth data.

#### 2.2.3 Soil and Plant Nutrients

We obtained soil organic nutrient data from the established decomposition experiment in October 2017 (Kolka et al. 2023). After three years' decomposition, organic soil with four depth ranges (0–10, 10–20, 20–30, and 30–40 cm) was sampled from hollow sites in October 2020. Total soil nitrogen (soil N, %), soil carbon (soil C, %), and soil phosphorus (soil P, %) at each depth were obtained from Kolka et al. (2023), with detailed methods described in Roth et al. (2023). Meanwhile, we obtained soil inorganic nutrients and soil temperature data from an ion‐exchange‐resin‐experiment, including plant available N (i.e., ammonium, NH_4_‐N in μg cm^−2^ year^−1^), P (i.e., phosphate, PO_4_‐P in μg cm^−2^ year^−1^), and NH_4_‐N:PO_4_‐P (Iversen et al. 2017). The ion‐exchange‐resin experiment was established in 2013, and there were two soil layers in the hollow site (0–10, 10–30 cm) and four soil layers in the hummock site (0–10, 10–30, 30–40, 40–60 cm). To include both warming and CO_2_ treatments, we used the accumulated yearly plant available inorganic nutrients data in 2018. To keep consistent soil moisture, nutrients and temperature, we always used the averaged data from hollow sites with a depth of 10–30 cm. For plant nutrients data, we obtained the measured mass‐ and area‐based leaf nitrogen (leaf N, % or N_leaf.A_ g m^−2^) in 2017 from previous studies (Dusenge et al. 2021, 2023), leaf phosphorus (leaf P, %), and stem nitrogen (stem N, %) from 2017 to 2020 (Phillips et al. 2021). Stem nutrient concentrations were measured using harvested current‐year branch rather than increment cores. The stem total N uptake (N_stem_ uptake, kg tree^−1^ yr.^−1^) was calculated based on the following formula: *N*_*stem*_ *uptake* = *N*_*stem*_ × *Biomass growth rate*. Meanwhile, we calculated soil and tissue C:N, C:P, and N:P ratios to evaluate nutrient allocation strategies. All soil and plant nutrient data were averaged per species per plot for further analysis.

### 2.3 Statistical Analyses at the Regional Scale

To assess how climate variables affect tree growth (BAI) in boreal Canada, we modeled log‐transformed BAI using a linear mixed‐effects model fitted with glmmTMB (Gaussian errors, identity link). Fixed effects included tree size (previous year basal area, BA_pre_), summer VPD, T_soil_, and SM, each interacting with species, and a smooth temporal component represented by the coefficients of a cubic regression‐spline basis for year. Random intercepts were fitted for site and tree, with site‐level random slopes for VPD, T_soil_, and SM. We included a first‐order autoregressive correlation structure to control for temporal autocorrelation using years within trees, and an exponential function to control for spatial autocorrelation using longitude and latitude. To accommodate heteroscedasticity, we modeled dispersion as a function of standardized prior basal area, allowing residual variance to vary with tree size. We used the previous summer VPD since it affected growth to a larger extent than the current year, and also due to the growth legacy effect (Girardin et al. 2024). Prior to analysis, we used the three climate variables (i.e., VPD, T_soil_, and SM) in the same model to address collinearity by applying the variance inflation factor (VIF) < 5. To compare the slope of each predictor, BA and climatic variables were log‐transformed and then standardized to a mean = 0 and a standard deviation of 1. We considered tree size impacts using basal area rather than tree age, since tree age was not available at the experimental scale to keep analyses consistent, and basal area was normally positively correlated with tree age (Song, Sass‐Klaassen, et al. 2021). Since T_air_ was highly correlated with VPD, we could not include both factors simultaneously. We thus ran an additional model to assess the effect of T_air_ on growth. The BAI‐climate model was fitted using the R package “glmmTMB” (Brooks et al. 2017).

### 2.4 Statistical Analyses at the Experimental Scale (SPRUCE)

#### 2.4.1 Growth‐Climate Relationships

To assess how climate variables affect tree growth at SPRUCE, we modeled log‐transformed BAI using a Tweedie generalized linear mixed model fitted with glmmTMB. Since there were true zero‐growth observations and right‐skewed positive BAI, we used a Tweedie distribution (Dunn and Smyth 2005). Fixed effects included tree size (BA_pre_), VPD, WT, and CO_2_ treatments and their interactions with species, and a smooth basis for year. We included random intercepts for plot and individual treeID (treeID nested within plot), and a dispersion submodel as a function of standardized BA_pre_, allowing residual variance to vary with tree size. To compare the slope of each predictor, BA and climatic variables were log‐transformed and then standardized to a mean = 0 and a standard deviation of 1. Since there is high collinearity among VPD, T_air_, and T_soil_, we could not include all three factors simultaneously; we thus ran two additional models to assess the effects of T_air_ and T_soil_ on growth (Figure S1). The R package ‘glmmTMB’ (Brooks et al. 2017) was used again to fit BAI‐climate relationships.

#### 2.4.2 Growth‐Trait or Trait–Trait Relationships

Because most traits were measured from one target individual per species per treatment, and some from multiple individuals, we used mean trait values per species per plot for all experimental analyses, such that each treatment enclosure was represented by one trait value per species and individual trees within an enclosure were not treated as independent replicates. To quantify the resource economics spectrum (RES), we performed three principal component analyses (PCAs) on 21 key functional traits across and within species, excluding traits with similar functions, such as K_s_ and wood anatomical traits, while retaining E_t_. To examine associations between growth, resource‐use strategies, and VPD within each species, we modeled the BAI as a function of the first two principal component scores, or alternatively, modeled the principal component scores as a function of VPD. The PCAs were performed using the R package “*factoextra*” (Kassambara and Mundt 2017). To explore bivariate growth‐trait relationships, we performed regression analyses for each species and CO_2_ treatment, with one of these trait values as the independent variable and BAI as the dependent variable. We therefore had three regressions per species for each trait variable: two under eCO_2_ and aCO_2_, and one under both CO_2_ treatments. Given the limited sample size per species and CO_2_ treatment (*n* = 4–5 under eCO_2_ or aCO_2_), we assessed significance using an exact two‐sided permutation test by enumerating all n! permutations of the response relative to the predictor. Permutation‐based *p*‐values were defined as the fraction of permutations yielding an absolute slope at least as large as the observed slope, using the permutations function within the R package “gtools” (Warnes et al. 2015). Meanwhile, we calculated the relative stem area growth rate (RGR, year^−1^) as RGR = (log BA_2021_ − log BA_2016_)/5 to account for the effect of initial tree size (Pommerening and Muszta 2016; Pommerening et al. 2023). Trait–RGR relationships were then examined as a sensitivity analysis. To investigate how functional traits were associated, we performed Pearson correlations per species.

#### 2.4.3 Trait‐VPD Relationships

To test the effects of VPD and CO_2_ on tree growth and trait values, we also performed linear regressions with either BAI or functional traits as the dependent variable, VPD, CO_2_, and species as the fixed effects. The interactions (VPD × Species × CO_2_, VPD × Species, or VPD × CO_2_) were considered in the models. To select the best model for each trait, we used the lowest adjusted Akaike information criterion corrected for small sample size (Dusenge et al. 2023) (AICc). Afterwards, the difference for the same trait values between eCO_2_ and aCO_2_ was calculated as Δ. To explore climate factors on the degree of CO_2_ impact, we did regressions of Δ against one of the climate variables. For all analyses, we log‐, cubic root‐, or square root‐transformed trait data when necessary to improve normality and homoscedasticity. All data were analyzed using R v.4.4.0 (R Core Team 2013).

## 3 Results and Discussion

### 3.1 Growth Responses to Environmental Drivers

As expected in our first two hypotheses, at the regional scale, VPD negatively and SM positively influenced growth in both larch and spruce (Figure 1b, Table S2). T_air_ had a significant but weak negative effect on growth in both larch (coefficient = −0.03, *p* < 0.001) and spruce (coefficient = −0.08, *p* < 0.001). Similarly, VPD had a significant but weak negative effect on BAI in both larch (coefficient = −0.03, *p* < 0.001) and spruce (coefficient = −0.05, *p* < 0.001). In contrast, growth at the experimental site was positively associated with VPD in larch (coefficient = 0.47, *p* < 0.001), but not in spruce (Figure 1c, Table S2). T_air_ was also positively correlated with growth in larch (coefficient = 0.49, *p* < 0.001; Figure S1) due to warming‐enhanced photosynthesis (Dusenge et al. 2023). However, eCO_2_ had no effects on aboveground growth, likely reflecting greater belowground carbon allocation (Iversen et al. 2008), as supported by a recent study on the same site (Hanson et al. 2025). Growth was unrelated to water table depth (WT), indicating non‐limiting water availability in this peatland (Dymond et al. 2019), consistent with the perennially high WT at the experimental site in a peat bog (Hanson et al. 2020).

Overall, larch exhibited a more sensitive and positive growth response to SM than spruce at the regional scale, and a more sensitive and positive response to VPD than spruce in the experimental site. Larch required a higher SM than spruce to achieve its more positive growth rates (Table S2). Additionally, larch exhibited growth responses that were negative to T_air_ at the regional scale but positive at the experimental scale. The decline in larch regional growth is due to its acquisitive strategy, since high stomatal conductance increases hydraulic failure risk (Figure 2), and warming‐induced high VPD likely triggers stomatal closure to reduce water loss (Jin et al. 2023), further limiting carbon uptake and growth. In comparison, spruce growth exhibited lower climate sensitivity because its conservative strategy maintains high hydraulic safety (Figure 2a). At the experimental site, consistently high WT, rapid warming‐induced soil nutrient release, T_air_‐ and VPD‐enhanced transpiration should support more nutrient, carbon, and water fluxes for growth (Figures 2, 3, 4, 5). Unlike at the experimental site, regional sites exhibited no significant BAI‐T_soil_ relationships (Figure 1b), likely because slower soil warming limits nutrient release (~0.05°C year^−1^ regionally vs. immediate treatment initiation up to +9°C experimentally), thereby constraining BAI‐T_soil_ responses and exacerbating negative BAI‐VPD relationships.

Comparison of the regional and experimental data reveals both distinctions and similarities in the growth sensitivity of larch and spruce. Growth sensitivities were lower at the regional scale than at the experimental scale, where the weak negative regional BAI‐VPD relationships shifted to positive. A key distinction is that regional sites experienced much weaker warming than the experimental site, highlighting warming intensity as an important driver of divergent sensitivities. In addition, the stronger positive effect of soil moisture relative to VPD on growth at the regional scale confirms soil moisture as a key climatic driver for both species. Despite these differences, some patterns were generalizable: larch consistently exhibited higher growth sensitivity to soil moisture regionally and to VPD experimentally, reflecting their positions along the RES and supported by concordant growth‐trait and trait–trait/VPD relationships (Figures 3, 4, 5).

**FIGURE 3:**
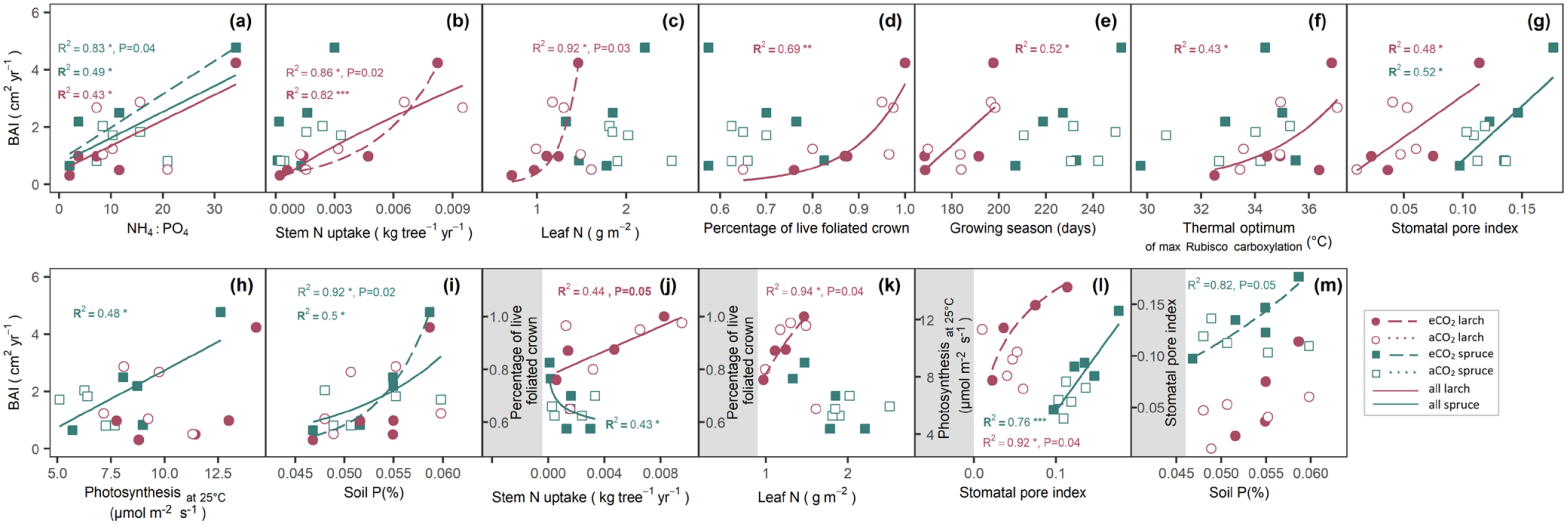
Basal area increment (BAI) relationships with nutrients and photosynthesis at the experimental site. (a–f) Larch BAI was generally associated with leaf‐ and whole‐tree nitrogen (N) uptake and allocation. (g–i) Spruce exhibited correlations with leaf‐level photosynthetic traits and phosphorus (P). (j–m) Mechanistic relationships between leaf and whole‐plant traits and fluxes that could underlie BAI responses. Red and teal indicate larch and spruce, respectively. Filled and open symbols indicate elevated (eCO_2_) and ambient CO_2_ (aCO_2_), respectively. Solid, long‐dashed, and dotted lines indicate significant regressions across CO_2_ treatments, under aCO_2_, and under eCO_2_, respectively. Bold, italic, and regular *R* ^2^ values correspond to regressions across CO_2_ treatments, under aCO_2_, and under eCO_2_, respectively. Only four plots were sampled for certain larch parameters under eCO_2_ due to limited sample size. When *n* = 4–5, permutation‐based *p*‐values are shown; they were computed using an exact two‐sided permutation test (all n! permutations), defined as the fraction of permutations with an absolute slope at least as large as the observed value. All regressions are linear, and data were log‐transformed when needed (nonlinear lines) to improve normality and homoscedasticity. *, *p* < 0.05; **, *p* < 0.01; ***, *p* < 0.001.

### 3.2 Mechanism Driving Divergent Growth Strategies for Nutrient and Carbon Uptake Across the RES

As expected in the third hypothesis, larch's acquisitive and spruce's conservative strategies along the RES were associated with their nutrient allocation, carbon assimilation, and water transport at the experimental site (Figure 2). We use this framework to investigate resource‐use strategies in response to environmental change. Larch growth was positively predicted by tree‐level carbon assimilation, water transport, and nitrogen availability, while spruce growth depended on leaf‐level carbon assimilation. Along the RES (Figure 2a), larch exhibited an acquisitive strategy characterized by strong carbon and water fluxes (i.e., high percentage of live foliated crown [PLFC], high stomatal conductance [g_s_], and high transpiration [E_t_]), whereas spruce was characterized by a conservative strategy with larger hydraulic safety margin (HSM), dense tissues (high leaf dry matter content [LDMC] and wood density [WD]) and higher water‐use efficiency (iWUE, the ratio of photosynthesis to g_s_). PCA revealed that tree‐level RES (PC1) increased larch growth and synergistically amplified the effect of summer VPD (Figure 2b,d,e). In contrast, spruce growth was governed by leaf‐level RES (PC2), integrating trade‐offs between photosynthesis and the decline in diurnal water potential. Spruce trees experiencing greater diurnal hydraulic drawdown operated at lower g_s_ and E_t_, reflecting hydraulic limitation and tighter stomatal control under water stress. Enhanced leaf‐level carbon assimilation along PC2 increased spruce growth, again facilitated by summer VPD (Figure 2c,g,h).

We then examined pairwise relationships between BAI and 45 functional traits, as well as soil nutrients. We used significant relationships to guide trait–trait analyses, focusing on traits significantly associated with BAI (Figure 3; Figure S2). While BAI in both species was positively associated with the soil NH_4_‐N:PO_4_‐P (Figure 3a), larch BAI was further supported by multiple indicators of increased soil nitrogen (N) availability under rising VPD and T_soil_ (Figure S3; Tables S3 and S4). For larch, warming‐enhanced soil N availability, resulting from increased N mineralization under rising T_soil_ (Jarvis and Linder 2000) and VPD‐induced moss mortality (Norby et al. 2019), supports its high N demand under an acquisitive strategy. Larch growth was further supported by enhanced photosynthesis at both whole‐tree and leaf‐level scales. Whole‐tree traits such as N uptake, N allocation to percentage of live foliated crown (PLFC), and growing season length were positively correlated with BAI (Figure 3b–e,j,k). At the leaf level, BAI was positively correlated with N allocation, and optimal temperatures for photosynthesis (T_optV_, Figure 3f). These shifts support a trait syndrome consistent with acquisitive resource use along the RES (Figure 2b), where high transpiration (E_t_), stomatal conductance (g_s_), and hydraulic conductivity (K_s_) facilitate increased nitrogen flux to leaves. Although water and nutrient uptake across root membranes involve distinct physiological processes, VPD‐induced increases in water flux (high K_s_ and E_t_) may enhance N delivery to roots via transpiration‐driven mass flow (Sands and Mulligan 1990) and subsequently facilitate N transport to leaves through the xylem. High N availability facilitates crown development via increased PLFC and thus photosynthetic capacity (Zhang et al. 2021), enhancing growth (Figure 3a–d,j,k) in line with stoichiometric requirements for growth (Salmon et al. 2021). Higher leaf N likely also promotes photosynthetic capacity (V_cmax_, maximum Rubisco carboxylation rate; Figure S4), as observed in temperature‐dependent shifts in photosynthetic capacity (Dusenge et al. 2023).

Spruce, by contrast, exhibited a more conservative strategy, with growth linked to leaf‐level photosynthesis and soil phosphorus (P) through stomatal anatomical shifts (Figure 3g–i,l,m). Increases in stomatal traits (Figure S2e–h) were positively correlated with BAI, especially SPI (surface area of stomata per leaf area; Figure 3g). Spruce growth was positively related to soil P and stomatal anatomical adjustments that enhance gas exchange for greater growth (Sack et al. 2003). Soil P uptake plays a critical role in SPI (Figure 3m) by supporting cell division and subsequent stomatal development (Khan et al. 2023). The importance of SPI for spruce may reflect its evergreen habit and long leaf lifespan for up to a decade (Reich et al. 2014), which favors strategic investments in leaf structure that persist for years. Coordinated shifts in soil P, leaf anatomy and photosynthetic rate further support the role of leaf‐level P‐mediated growth in spruce.

These contrasting strategies position larch and spruce at opposite ends of the RES, with larch relying on short‐lived tissues that support high tree‐level carbon and water flux, and spruce prioritizing long‐term investment in leaf structure and leaf‐level gas exchange efficiency (Figure 2). This pattern was also observed when we used the relative growth rate (Figure S5). The acquisitive strategy makes larch respond fast to climate change with high sensitivity, such as due to pulses of nutrients at the experimental site or favorable climate conditions regionally that could allow larch to become more dominant than spruce in boreal forests. However, spruce's location in the conservative RES range could offset larch's faster growth by conferring greater longevity.

### 3.3 Mechanism Driving Divergent Growth Strategies for Water Uptake Across the RES

Both larch and spruce exhibited positive correlations between BAI and transpiration (E_t_, Figure 4a). Increasing E_t_ was driven by larger PLFC in larch and higher g_s_ in spruce (Figure 4b,c), which fed back on BAI through enhanced photosynthesis associated with larger crowns in larch and higher g_s_ in spruce; the latter increasing multiple indices of leaf‐level photosynthesis (Figure 4d).

**FIGURE 4:**
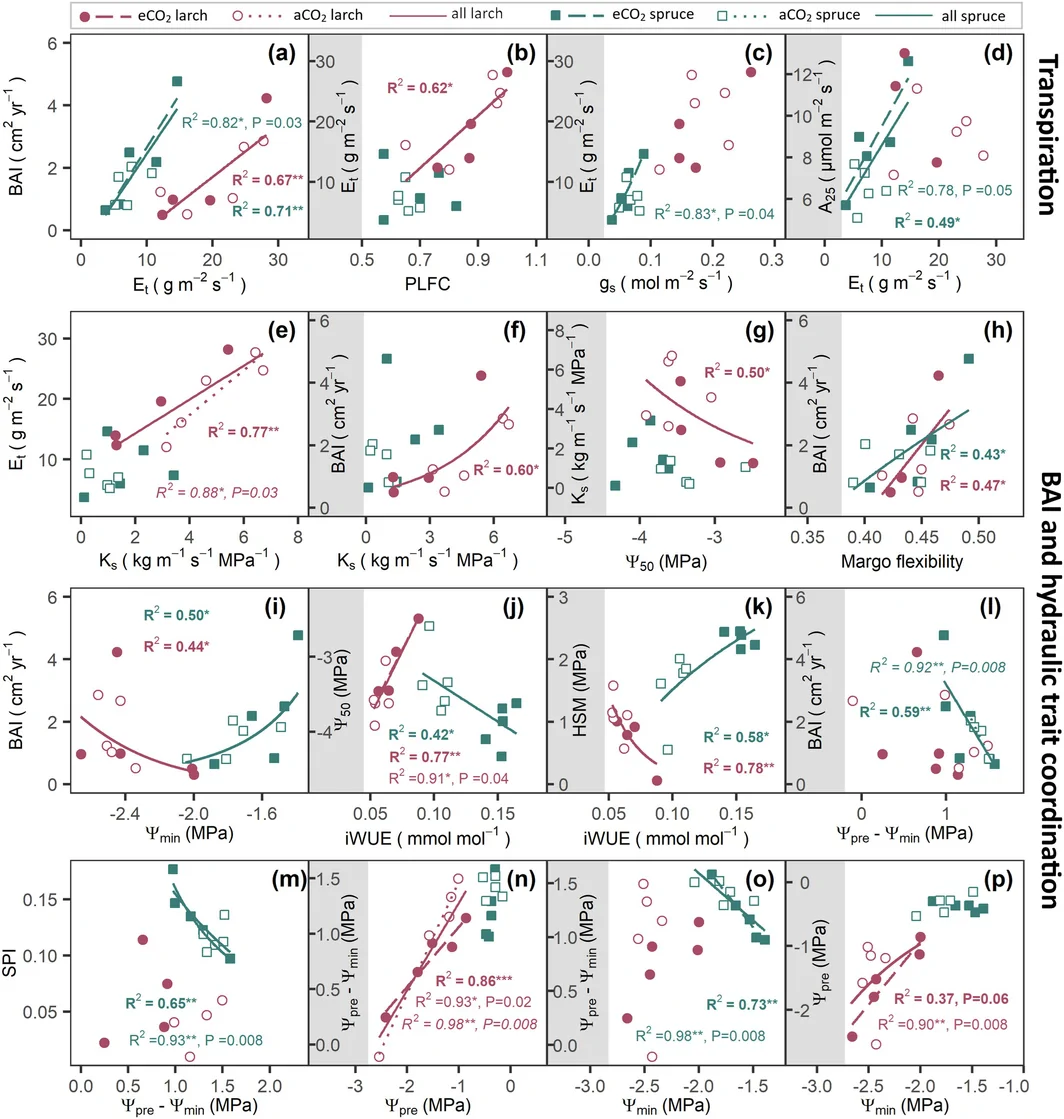
Relationships between basal area increment (BAI), transpiration and hydraulic trait coordination at the experimental site. (a–d) BAI is affected by transpiration (E_t_). (a–d) BAI increased with E_t_ for both species through whole‐tree‐level percentage of live foliated crown (PLFC) for larch and leaf‐level stomatal conductance (g_s_) and photosynthesis (A_25_) for spruce. (e–g) High hydraulic conductivity (K_s_) increased E_t_ and BAI, with even high embolism resistance (more negative Ψ_50_) for larch. (h) Large margo flexibility (MF) increased BAI due to high K_s_ and E_t_ without greater embolism vulnerability for both species. (i) BAI decreased and increased with Ψ_min_ for larch and spruce, respectively. (j, k) Divergent hydraulic safety (Ψ_50_; hydraulic safety margin, HSM) responses to water‐use efficiency (iWUE). (l, m) The difference between predawn and minimum water potential (Ψ_pre_‐Ψ_min_) decreased BAI and SPI in spruce. (n–p) The anisohydric and isohydric behavior for larch and spruce, respectively. Red and teal indicate larch and spruce, respectively. Filled and open symbols indicate elevated (eCO_2_) and ambient CO_2_ (aCO_2_), respectively. Solid, long‐dashed, and dotted lines indicate significant regressions across CO_2_ treatments, under aCO_2_, and under eCO_2_, respectively. Bold, italic, and regular *R* ^2^ values correspond to regressions across CO_2_ treatments, under aCO_2_, and under eCO_2_, respectively. Only four plots were sampled for certain parameters of larch under eCO_2_ due to limited sample size. When *n* = 4–5, permutation‐based *p*‐values are shown; they were computed using an exact two‐sided permutation test (all n! permutations), defined as the fraction of permutations with an absolute slope at least as large as the observed value. All regressions are linear, and data were log‐transformed when needed (nonlinear lines) to improve normality and homoscedasticity. *, *p* < 0.05; **, *p* < 0.01; ***, *p* < 0.001.

Positive BAI‐E_t_ relationships reflect contrasting hydraulic strategies balancing hydraulic efficiency‐safety trade‐offs. In larch, high E_t_ was achieved by K_s_ (Figure 4e), resulting in an associated relationship between BAI and K_s_ (Figure 4f). Both species achieved high K_s_ and E_t_ via large tracheids (D_h_), large pits in larch, and large lumen fraction for conductance in spruce (Figure S6a–c), enhancing axial and lateral water transport capacity (Choat et al. 2008; Pittermann et al. 2006), respectively. These shifts often typically reduce hydraulic safety (Song, Poorter, et al. 2021); yet larch exhibited a surprising increase in xylem resistance to embolism (more negative Ψ_50_) with increasing K_s_, while spruce held a constant K_s_‐Ψ_50_ relationship (Figure 4g). This counter‐intuitive result lies in positive relationships between BAI and margo flexibility (MF; Figure 4h), since high MF can enhance both embolism resistance (Delzon et al. 2010; Song, Poorter, et al. 2021) and hydraulic transport (Hacke et al. 2004) (Figure S6d). Larch also increased wall thickness to resist implosion (Hacke et al. 2001) for a lower Ψ_50_ (Figure S6e), whereas spruce maintained hydraulic safety via shifts in smaller D_h_ and SPI to minimize xylem embolism and foliar water loss (Figure S6f–h).

Species‐specific coordination of water potential, gas exchange, and growth reflects contrasting hydraulic strategies. Relationships between BAI, E_t_, and wood anatomy were consistent with the species‐specific regulation of leaf‐level water potential and gas exchange, mediated by anatomical trait (SPI) and iWUE. BAI decreased with minimum water potential (Ψ_min_) in larch but increased in spruce (Figure 4i), showing larch's acquisitive strategy of risking hydraulic failure for growth versus spruce's conservative strategy of enhancing transpiration while maintaining high hydraulic safety margins (HSMs). Spruce tightened coordination of iWUE with Ψ_50_ toward greater safety, whereas larch had low iWUE at low Ψ_50_ (Figure 4j). Consequently, HSM and iWUE were negatively related in larch (Figure S4) but positively in spruce (Figure 4k; Figure S7). Spruce BAI, SPI, and Ψ_pre_‐Ψ_min_ were correlated (Figure 4l,m), suggesting tight regulation of water potential gradients. Larch Ψ_pre_‐Ψ_min_ was controlled by Ψ_pre_ (Figure 4n) and spruce by Ψ_min_ (Figure 4o), consistent with soil‐driven Ψ_pre_ in larch versus stomatal‐regulated Ψ_min_ in spruce. These differences reflect species‐specific degrees of anisohydry (Figure 4p), which shift under increasing stress within (Charrier et al. 2018; Hochberg et al. 2018) and across species (Martínez‐Vilalta et al. 2014). Greater anisohydry supports the acquisitive water‐use strategy in larch, while stronger isohydry supports the conservative hydraulic safety strategy in spruce.

### 3.4 Hydraulic Strategies in Response to Rising VPD

Although BAI was positively correlated with VPD across species (Figure 5a), the trait‐VPD relationships (Figure 5b–l, Figure S8, Table S5) revealed contrasting hydraulic strategies in larch and spruce, reflecting trade‐offs to balance growth and hydraulic safety through coordinated photosynthetic, hydraulic, and anatomical traits. Both species exhibited trait shifts associated with rising VPD, such as positive correlations between A_25_ and VPD (Figure 5b), partly aided by higher g_s_ (Figure 5c). These gas exchange gains reduced iWUE in larch but left spruce unchanged (Figure 5d). Whole‐plant photosynthesis also increased with VPD via longer growing seasons in both species and larger crowns in larch (Figure 5e,f). Increased leaf‐ and whole‐tree water demand was met through higher K_s_ in both species (Figure 5g), with larch exhibiting greater transpiration than spruce (Figure 5h). Larch also exhibited more negative Ψ_pre_ with rising VPD while maintaining stable Ψ_min_ (Figure 5i), indicating greater soil water extraction, as reflected by a narrower Ψ_pre_‐Ψ_min_ (Figure 5j). Spruce exhibited less negative Ψ_50_ with rising VPD, while larch appeared stable or even exhibited more negative Ψ_50_ under eCO_2_ (Figure 5k). HSMs were unchanged in both species (Figure 5l). Notably, larch has a lower HSM than spruce due to its riskier approach with higher g_s_ and E_t_ under rising VPD, which allows larch to better capitalize on increased temperature, nutrients, but requires hydraulic shifts to maximize water transport with minimal loss in embolism resistance, supporting our last hypothesis.

**FIGURE 5:**
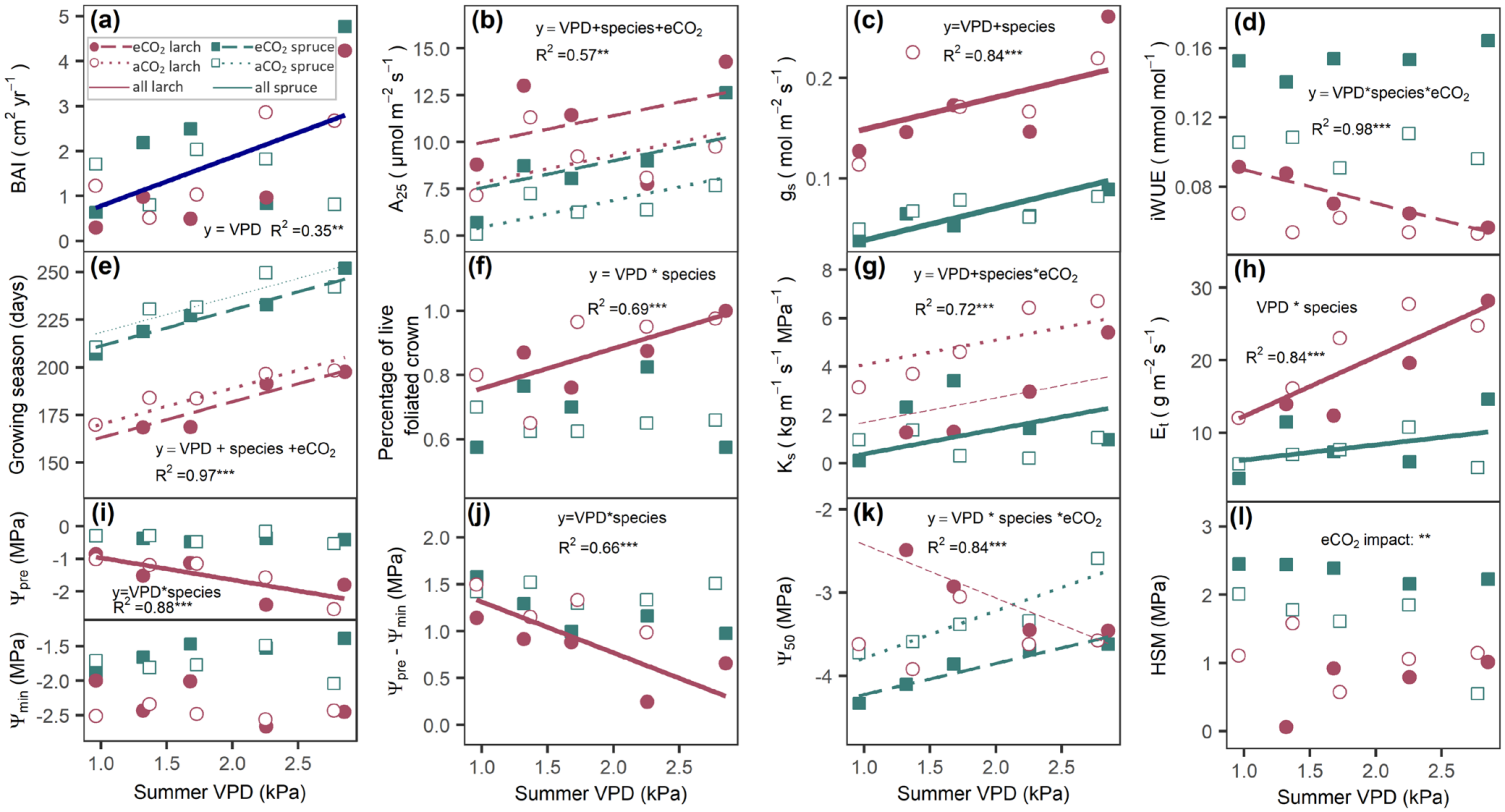
Impacts of vapor pressure deficit (VPD) and CO_2_ on physiological traits related to carbon assimilation and hydraulics. (a) Basal area increment (BAI) is positively associated with increasing vapor pressure deficit (VPD). (b–f) Carbon assimilation‐related trait shifts. (g–l) Hydraulic trait shifts. A_25_, photosynthesis; g_s_, stomatal conductance; iWUE, water‐use efficiency; PLFC, whole‐tree percentage of live foliated crown; K_s_, hydraulic conductivity; E_t_, transpiration; Ψ_pre_, predawn water potential; Ψ_min_, minimum water potential; Ψ_50_, xylem potential inducing 50% loss of hydraulic conductivity; HSM, hydraulic safety margin. Red and teal indicate larch and spruce, respectively. Filled and open symbols indicate elevated (eCO_2_) and ambient CO_2_ (aCO_2_), respectively. Solid, long‐dashed, and dotted lines indicate significant regressions across CO_2_ treatments, under aCO_2_, and under eCO_2_, respectively. Equations for the best‐fitting model, based on AICc (Table S6) and *R* ^2^, are shown. Significant differences in interceptions are indicated by separate regression lines within each trait. For full statistical results, see Table S5. *, *p* < 0.05; **, *p* < 0.01; ***, *p* < 0.001.

Larch's smaller VPD‐induced growth reductions regionally (Figure 1b) and stronger growth increases experimentally (Figure 1c) reflect its acquisitive hydraulic strategy that maximizes nutrient and carbon uptake at the cost of hydraulic safety. This strategy involved coordinated increases in g_s_ (Figure 5c) and K_s_ (Figure 5g) via anatomical shifts including larger tracheids, pits, and margo flexibility (Figure S6a,b,d), enabling high transpiration (Figure 5h). In contrast, spruce exhibited limited wood anatomical shifts beyond increases in tracheid density and lumen fraction (Figure S2i,j), which could increase water flow given the lack of change in D_h_. Instead, spruce exhibited substantial shifts in stomatal anatomy with rising VPD (Table S5), reflecting a conservative strategy based on stomatal regulation rather than xylem structural shifts to maintain gas exchange and hydraulic safety.

The iWUE responses further highlighted divergent coordination between carbon assimilation and hydraulic safety. Larch's iWUE declined with rising VPD (Figure 5d) and was negatively correlated with g_s_ (*r* = −0.65, *n* = 10, *p* = 0.04), reflecting high‐risk, acquisitive strategies that prioritized carbon assimilation through high transpiration, unlike typical conifer responses (Leonardi et al. 2012). Spruce, in contrast, maintained conservative water‐saving strategies with high iWUE and parallel shifts in photosynthesis and g_s_ under rising VPD (Figure 5b–d). Spruce's relatively stable g_s_ in comparison to larch (Warren et al. 2021) limited its plasticity but favored long‐term investments in leaf structure, resulting in higher carbon gain without sacrificing HSM via wood anatomical shifts (Figure S6f). The evergreen habit and high leaf construction costs (i.e., higher LDMC and LMA than larch; Table S5) further supported the conservative strategy in spruce. These divergent patterns extended to growth responses. Larch BAI increased as iWUE declined, while spruce BAI remained stable regardless of iWUE (Figure S2d), suggesting contrasting trade‐offs between carbon gain and water‐use efficiency. Hydraulic trait shifts reinforced this differentiation. Larch exhibited declining HSM with increasing iWUE, and spruce the opposite (Figure 4k), indicative of trait coordination wherein HSM increased with K_s_, g_s_, and declined with iWUE in larch, while spruce exhibited a more conservative approach, with iWUE increases being indicative of greater hydraulic tension (Ψ_pre_‐Ψ_min_) and lower BAI (Figure 4l). In spruce, this was manifest via the relationships between iWUE and BAI with SPI (Figures 3g, 4m). Our results suggest that warming and drying, even in wet peatlands, could increasingly favor conservative species like spruce while compromising acquisitive species such as larch, with long‐term consequences for forest composition and function under climate change.

### 3.5 Physiological Mechanisms of Growth Responses to eCO_2_

Both species exhibited shared trait shifts to eCO_2_, including higher A_25_ and iWUE (Figure 5b,d), lower N and V_cmax_, and shorter growing seasons via earlier green‐down (Figsure S8 and S9). However, hydraulic responses diverged markedly, strengthening contrasting resource‐use strategies. Larch showed stable HSMs under eCO_2_, whereas spruce exhibited larger HSMs owing to reduced Ψ_50_ (Figure 5i,k,l), suggesting reinforced embolism resistance. Larch's acquisitive strategy emphasizes early‐season carbon gain under eCO_2_ (Linkosalo et al. 2017) (Figure 2f) but relies on earlier senescence to conserve water, as reflected in lower K_s_ (Figure 5g). Although peatlands are typically wet in spring, late‐summer soil drying and rising VPD likely trigger water stress and earlier larch senescence. By contrast, spruce's conservative strategy combines enhanced photosynthesis with greater hydraulic safety, supporting faster growth and greater drought resistance with higher iWUE (Figure 5d). Although regional (García‐Valdés et al. 2021; Johnson and Brodribb 2023) and global (Choat et al. 2018) studies reported trade‐offs between growth and drought resistance in dry areas, a recent study in boreal and temperate forests found positive relationships in conservative species (Augusto et al. 2025) consistent with our findings.

Trait responses to rising VPD further emphasized these strategies, as analyzed in trait shift values between eCO_2_ and aCO_2_ (Δ). In larch, eCO_2_‐induced reductions in growing season length (Δ < 0) and increases in iWUE (Δ > 0) weakened with rising VPD (Figure S9a,b), indicating limited acclimation under atmospheric stress. In contrast, spruce exhibited multiple trait shifts under eCO_2_ that strengthened with rising VPD toward greater leaf‐level carbon gain and more embolism‐resistant xylem (Figure 2h,i; Figure S9c–f). Together, these findings highlight limited adjustment in acquisitive larch versus sustained assimilation and hydraulic safety in conservative spruce under elevated eCO_2_ and warming.

## 4 Conclusions

Our study highlights the importance of contrasting functional strategies in shaping the structure and carbon dynamics of boreal peatland forests under climate change (Figure 6). Under future rising VPD and elevated CO_2_, both larch and spruce maintain growth via distinct physiological mechanisms linked to their positions on the RES. Larch, with its acquisitive strategy, supports rapid growth via enhanced nutrient uptake and water use, but remains vulnerable to drought‐induced hydraulic failure due to lower safety margins. In contrast, spruce exhibits a conservative strategy that maintains greater hydraulic safety and benefits from CO_2_ fertilization, buffering future drought stress. However, prolonged exposure to elevated CO_2_ and warming potentially limits growth in acquisitive conifers, while enhancing both growth and survival in conservative conifers, even in peatland systems. These findings provide mechanistic insights into predicting ecosystem carbon feedbacks in a warmer, drier world.

**FIGURE 6:**
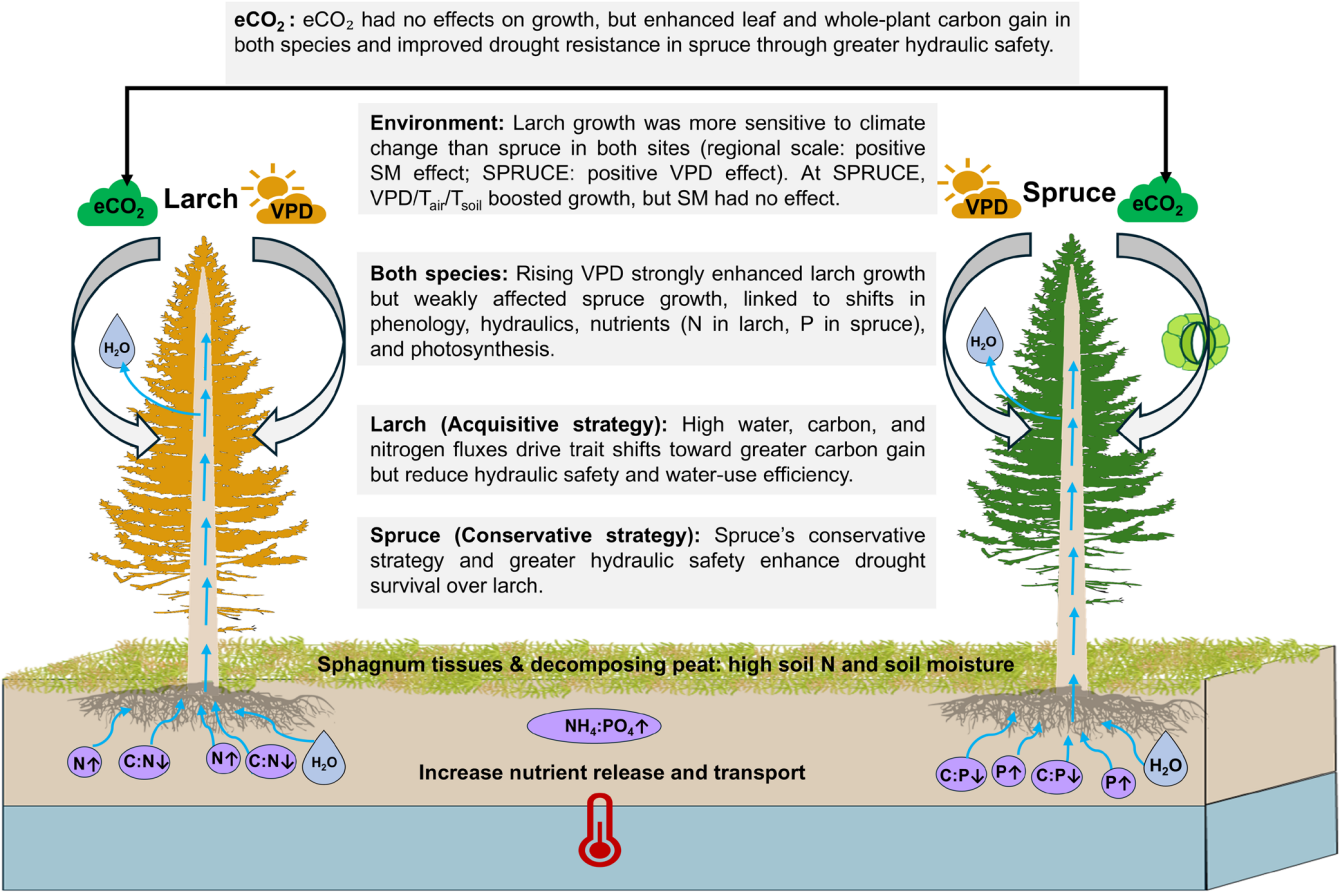
Proposed mechanisms underlying growth responses to rising VPD and eCO_2_ in the acquisitive larch and conservative spruce. Although eCO_2_ did not affect growth, it enhanced carbon assimilation via increased photosynthesis in both species. The hydraulic response to eCO_2_ diverged, with stable hydraulic safety in larch and higher safety in spruce. Larch growth was always more sensitive to climate change than spruce at both scales, with a stronger positive response to SM than to VPD at the regional scale and a stronger positive response to VPD/T_air_/T_soil_ than to SM at the experimental scale. At the experimental site, T_soil_ boosted growth, but soil moisture had no effect. Overall, the acquisitive strategy of larch supports high transpiration and nitrogen uptake, facilitated by anatomical shifts, increased VPD, and soil warming. In contrast, the conservative strategy of spruce maintains greater hydraulic safety than larch, contributing to higher survival and lower climate sensitivity under future warming.

## Author Contributions

**Yanjun Song:** conceptualization, methodology, data curation, formal analysis, validation, investigation, writing – original draft, writing – review and editing, visualization. **Alexandria Pivovaroff:** writing – review and editing, supervision, funding acquisition, conceptualization. **Mahmuda Islam:** writing – review and editing, data curation. **Martin P. Girardin:** methodology, resources, writing – review and editing, formal analysis. **Alana Chin:** methodology, writing – review and editing, data curation. **Hervé Cochard:** writing – review and editing, methodology. **José M. Torres‐Ruiz:** methodology, writing – review and editing. **José A. Medina‐Vega:** writing – review and editing, data curation, formal analysis, investigation. **Sylvain Delzon:** writing – review and editing, methodology. **Xiaohan Yin:** writing – review and editing, data curation. **Xiao Jing Guo:** methodology, data curation. **Mirindi E. Dusenge:** methodology, data curation, writing – review and editing. **Jeffrey M. Warren:** writing – review and editing, project administration, resources, data curation, funding acquisition, methodology. **Paul J. Hanson:** writing – review and editing, project administration, resources, data curation, methodology, funding acquisition. **Peter B. Reich:** methodology, writing – review and editing, investigation. **Andrew D. Richardson:** writing – review and editing, resources, investigation, methodology, data curation, formal analysis. **Lingcheng Li:** methodology, writing – review and editing. **Avni Malhotra:** methodology, writing – review and editing. **Verity G. Salmon:** methodology, writing – review and editing. **Nate G. McDowell:** conceptualization, supervision, writing – review and editing, funding acquisition, investigation, formal analysis.

## Funding

This work was supported by the U.S. Department of Energy, No. DE‐AC05‐00OR22725. National Science Foundation, IOS‐2220865.

## Conflicts of Interest

The authors declare no conflicts of interest.

## Supporting information


**Table S1:** Abbreviations of growth and the related 45 functional traits, and soil nutrients in this study.
**Table S2:** Summary of the fitted generalized linear mixed models of BAI.
**Table S3:** *R* ^2^ explained by bivariate relationships between BAI, VPD, T_soil_, WT and nutrients. The BAI group denotes the impacts of soil and plant nutrients on BAI. VPD, T_soil_, and soil water table depth (WT) groups denote their relationships with soil and plant nutrients. When *n* = 4–5, permutation‐based *p*‐values were computed using an exact two‐sided permutation test (all n! permutations). The plus sign in green (+) and minus sign in grey (−) denote positive and negative relationships, respectively. + or −, *p* < 0.05; + + or − −, *p* < 0.01; + + + or − − −, *p* < 0.001. The plus or minus signs in blue mean *p* = 0.06.
**Table S4:** *R* ^2^ explained by bivariate significant relationships between soil nutrients and plant nutrients. The plus sign in green (+) and minus sign in grey (−) denote positive and negative relationships, respectively. + or −, *p* < 0.05; + + or **− −**, *p* < 0.01; **+ + +** or **− − −**, *p* < 0.001. The plus or minus signs in blue mean *p* = 0.06. *R* ^2^ for nonsignificant relationships were not shown. For trait abbreviations, see Table S1.
**Table S5:** Summary of an analysis of covariance (ANCOVA), where summer VPD is a covariate, CO_2_ treatment and species as fixed factors, testing the relationship between trait values and summer VPD. The regression was selected based on the lowest adjusted Akaike information criterion (AICc) in Extended Data Table 8. Non‐significant regressions were not shown.
**Table S6:** Model comparison between models with interaction of the main treatment effects and models with only main treatment effects using adjusted Akaike information criterion (AICc). For trait abbreviations, see Table S1.
**Figure S1:** Effects of climate variables on basal area growth for larch and spruce at the SPRUCE Ecosystem experiment from 2017 to 2021. (a) Growth‐climate relationships for air temperature (T_air_), soil water table depth from surface (WT), and elevated CO_2_ (eCO_2_). (b) Growth‐climate relationships for T_soil_, WT and eCO_2_. Tree size indicates basal area. The x axes indicate the values of standardized coefficients with error bars (95% confidence intervals), and the y axes indicate climate and tree size factors. Dashed lines indicate that the standardized coefficients are not different from zero.
**Figure S2:** Significant relationships between BAI and physiological traits. Red and teal indicate larch and spruce, respectively. Filled and open symbols indicate elevated (eCO_2_) and ambient CO_2_ (aCO_2_), respectively. Solid, long‐dashed, and dotted lines indicate significant regressions across CO_2_ treatments, under aCO_2_, and under eCO_2_, respectively. Bold, italic, and regular *R* ^2^ values correspond to regressions across CO_2_ treatments, under aCO_2_, and under eCO_2_, respectively. When *n* = 4–5, permutation‐based *p*‐values are shown; they were computed using an exact two‐sided permutation test (all n! permutations), defined as the fraction of permutations with an absolute slope at least as large as the observed value. All regressions are based on linear regressions, and data were log‐transformed when needed (non‐linear lines) to improve normality and homoscedasticity. *, *p* < 0.05; **, *p* < 0.01; ***, *p* < 0.001. For trait abbreviations, see Table S1.
**Figure S3:** Climate‐driven soil nutrients affect tree growth. (a–f) climate variables including VPD, T_soil_, and soil water table depth affect soil and plant nutrients. Black symbols indicate eCO_2_ and grey symbols indicate aCO_2_. Black, grey, and red lines indicate the regression for eCO_2_, aCO_2_, and all CO_2_ treatments, respectively. (g–k) soil and leaf nutrients impact tree growth; red and teal indicate larch and spruce, respectively. Filled and open symbols indicate elevated (eCO_2_) and ambient CO_2_ (aCO_2_), respectively. Solid, long‐dashed, and dotted lines indicate significant regressions across CO_2_ treatments, under aCO_2_, and under eCO_2_, respectively. Bold, italic, and regular *R* ^2^ values correspond to regressions across CO_2_ treatments, under aCO_2_, and under eCO_2_, respectively. NH_4_:PO_4_, soil inorganic nitrogen to phosphorus ratio; soil C:N, soil organic carbon to nitrogen ratio; soil C:P, soil organic carbon to phosphorus ratio. When *n* = 4–5, permutation‐based *p‐values* are shown; they were computed using an exact two‐sided permutation test (all n! permutations). All regressions are based on linear regressions, and data were log‐transformed when needed (non‐linear lines) to improve normality and homoscedasticity. *, *p* < 0.05; **, *p* < 0.01; ***, *p* < 0.001.
**Figure S4:** Heatmap showing pairwise Pearson correlation coefficients (*r*) among 35 traits within larch (*n* = 9). Cell color indicates the magnitude and direction of *r*. Warm (red) shades indicate positive correlations and cool (blue) shades indicate negative correlations. Rows and columns were hierarchically clustered (complete linkage) using 1 − |*r*| as the distance metric. For trait abbreviations, see Table S1.
**Figure S5:** Significant relationships between relative growth rate (RGR) and physiological traits. Red and teal indicate larch and spruce, respectively. Filled and open symbols indicate elevated (eCO_2_) and ambient CO_2_ (aCO_2_), respectively. Solid, long‐dashed, and dotted lines indicate significant regressions across CO_2_ treatments, under aCO_2_, and under eCO_2_, respectively. Bold, italic, and regular *R* ^2^ values correspond to regressions across CO_2_ treatments, under aCO_2_, and under eCO_2_, respectively. When *n* = 4–5, permutation‐based *p*‐values are shown; they were computed using an exact two‐sided permutation test (all n! permutations), defined as the fraction of permutations with an absolute slope at least as large as the observed value. All regressions are based on linear regressions, and data were log‐transformed when needed (non‐linear lines) to improve normality and homoscedasticity. *, *p* < 0.05; **, *p* < 0.01; ***, *p* < 0.001. For trait abbreviations, see Table S1.
**Figure S6:** Wood anatomical traits determine hydraulics. (a–d) hydraulic efficiency. (e–h) hydraulic safety. Red and teal indicate larch and spruce, respectively. Filled and open symbols indicate elevated (eCO_2_) and ambient CO_2_ (aCO_2_), respectively. Solid, long‐dashed, and dotted lines indicate significant regressions across CO_2_ treatments, under aCO_2_, and under eCO_2_, respectively. Bold, italic, and regular *R* ^2^ values correspond to regressions across CO_2_ treatments, under aCO_2_, and under eCO_2_, respectively. When *n* = 4, permutation‐based *p*‐values are shown; they were computed using an exact two‐sided permutation test (all n! permutations). All regressions are based on linear regressions, and data were log‐transformed when needed (non‐linear lines) to improve normality and homoscedasticity. *R* ^2^ for each regression line is shown. *, *p* < 0.05; **, *p* < 0.01; ***, *p* < 0.001. For trait abbreviations, see Table S1.
**Figure S7:** Heatmap showing pairwise Pearson correlation coefficients (*r*) among 35 traits within spruce (*n* = 10). Cell color indicates the magnitude and direction of *r*. Warm (red) shades indicate positive correlations and cool (blue) shades indicate negative correlations. Rows and columns were hierarchically clustered (complete linkage) using 1 − |*r*| as the distance metric. *, 0.01 < *p* < 0.05; **, 0.001 < *p* < 0.01; ***, *p* < 0.001. For trait abbreviations, see Table S1.
**Figure S8:** Impacts of vapor pressure deficit (VPD) and CO_2_ on physiological traits related to hydraulics and carbon assimilation. Red and teal indicate larch and spruce, respectively. Filled and open symbols indicate elevated (eCO_2_) and ambient CO_2_ (aCO_2_), respectively. Solid, long‐dashed, and dotted lines indicate significant regressions across CO_2_ treatments, under aCO_2_, and under eCO_2_, respectively. Bold, italic, and regular *R* ^2^ values correspond to regressions across CO_2_ treatments, under aCO_2_, and under eCO_2_, respectively. Equations for the best‐fitting model, based on AICc and *R* ^2^, are shown. Significant differences in interceptions are indicated by separate regression lines within each trait. For full statistical results, see Table S5. *, *p* < 0.05; **, *p* < 0.01; ***, *p* < 0.001. For trait abbreviations, see Table S1.
**Figure S9:** Impacts of VPD on trait differences between eCO_2_ and aCO_2_ (Δ, see equation in panel a). (a–b) significant impacts of VPD on Δ for larch. (c–f) impacts of VPD on Δ for spruce. Red and teal indicate larch and spruce, respectively. Filled and open symbols indicate elevated (eCO_2_) and ambient CO_2_ (aCO_2_), respectively. Solid, long‐dashed, and dotted lines indicate significant regressions across CO_2_ treatments, under aCO_2_, and under eCO_2_, respectively. Due to the limited number of trees available to sample in some experimental treatments, only four plots were sampled for certain parameters of larch under eCO_2_ (dashed lines). All regressions are linear, and data were log‐transformed when needed (nonlinear lines) to improve normality and homoscedasticity. *R* ^2^ for each regression line is shown. *, *p* < 0.05; **, *p* < 0.01; ***, *p* < 0.001.

## Data Availability

Raw tree‐ring data from regional sites are available at the TreeSource repository (https://treesource.rncan.gc.ca/en/treerings). Photosynthesis‐related data are available at https://doi.org/10.6084/m9.figshare.22645030. Wood anatomy data are available at https://doi.org/10.15485/3030079. Additional datasets were obtained from the SPRUCE project website, including leaf phenology (https://doi.org/10.25581/spruce.108/1996813), growth (https://doi.org/10.25581/spruce.051/1433836), plant nutrients (https://doi.org/10.25581/spruce.056/1455138; https://doi.org/10.25581/spruce.090/1780604), soil organic nutrients (https://doi.org/10.25581/spruce.111/1991516), soil inorganic nutrients (https://doi.org/10.3334/CDIAC/spruce.036), environment data (https://doi.org/10.3334/CDIAC/spruce.032), and water potential (https://doi.org/10.25581/spruce.080/1615776).
