# Supplementary material for: Conservative Spruce Outperforms Acquisitive Larch Under Warming and CO2
Source: Glob Chang Biol. 2026 Oct 24;32(10):e71157. doi: 10.1111/gcb.71157 (PMC13695017; doi:10.1111/gcb.71157)
Supplement: Supplementary file 1 [file GCB-32-e71157-s001.docx]

**Supplementary Tables and Figures for**

**Conservative boreal spruce outperforms acquisitive larch under warming and CO_2_**

Tables S1-S6

Figs. S1-S9

**Supplementary Table 1** Abbreviations of growth and the related 45 functional traits, and soil nutrients in this study.

|  | **Abbreviations** | **Description** | **Unit** |
| --- | --- | --- | --- |
| Growth | BAI | Stem basal area growth rate | cm^2^ yr^-1^ |
| Hydraulics (7) | K_s_ | The maximum specific hydraulic conductivity | Kg m^-2^ s^-1^ MPa^-1^ |
|  | E_t_ | Transpiration, defined as sap flow density | g m^-2^ s^-1^ |
|  | Ψ_50_ | Averaged xylem pressure inducing 50% loss of hydraulic conductivity | MPa |
|  | Ψ_pre_^a^ | Predawn water potential | MPa |
|  | Ψ_min_^a^ | Minimum water potential | MPa |
|  | Ψ_pre_-Ψ_min_^a^ | The difference between predawn and minimum water potential | MPa |
|  | HSM | The difference between Ψ_min_ and Ψ_50_ | MPa |
| Wood anatomy (13) | WD | Wood density | g cm^-3^ |
|  | D_h_.e | Earlywood hydraulic diameter | µm |
|  | D_h_.l | Latewood hydraulic diameter | µm |
|  | TD.e | Earlywood tracheid density | mm^-2^ |
|  | TD.l | Latewood tracheid density | mm^-2^ |
|  | T_w_.e | Earlywood wall thickness | µm |
|  | T_w_.l | Latewood wall thickness | µm |
|  | LF.e | Earlywood lumen fraction as the ratio of lumen area to xylem area | - |
|  | LF.l | Latewood lumen fraction as the ratio of lumen area to xylem area | - |
|  | DPM | Pit membrane diameter | µm |
|  | DT | Torus diameter | µm |
|  | DPA | Pit aperture diameter | µm |
|  | MF | Margo flexibility | - |
| Photosynthesis  related traits (20) | PLFC | Percentage of live foliated crown |  |
|  | GSL^b^ | Growing season length | days |
|  | Green-up^b^ | The day of year for leaf green up | DOY |
|  | Green-down^b^ | The day of year for leaf green down | DOY |
|  | A_25_^c^ | Net photosynthetic rate estimated at 25 ℃ | μmol m^-2^ s^-1^ |
|  | iWUE^c^ | Water-use efficiency | mmol mol^-1^ |
|  | T_optV_^c^ | Thermal optimum of maximum Rubisco carboxylation rate | ℃ |
|  | g_s_^c^ | Stomatal conductance measured at 25 ℃ | mol m^-2^ s^-2^ |
|  | V_cmax_^c^ | Maximum Rubisco carboxylation rate at leaf temperature of 25 ℃ | μmol m^-2^ s^-1^ |
|  | SLA | Specific leaf area | cm^2^ g^-1^ |
|  | LDMC | Leaf dry matter content | g g^-1^ |
|  | Aba.Epidermal | Abaxial epidermal cell length | μm |
|  | Aba.Guard | Abaxial guard cell length | μm |
|  | Aba.SD | Abaxial stomatal density | # mm^-2^ |
|  | Aba.SPI | Abaxial stomatal pore index | - |
|  | Ada.Epidermal | Adaxial epidermal cell length | μm |
|  | Ada.Guard | Adaxial guard cell length | μm |
|  | Ada.SD | Adaxial stomatal density | # mm^-2^ |
|  | Ada.SPI | Adaxial stomatal pore index | - |
|  | SPI | Total stomatal pore index | - |
| Leaf and stem  nutrients (5) | N_leaf.A_^d^ | Leaf nitrogen concentration per leaf area | g m^-2^ |
|  | leaf N^d^ | Leaf nitrogen concentration per dry mass | % |
|  | leaf P^e^ | Leaf phosphorus per dry mass | % |
|  | stem N^e^ | Stem nitrogen per dry mass | % |
|  | N_stem_ uptake | The stem total N uptake=stem N*Biomass growth rate | kg tree^-1^ yr^-1^ |
| Soil nutrients (9) | soil C^f^ | Total organic soil carbon per dry mass | % |
|  | soil N^f^ | Total organic soil nitrogen per dry mass | % |
|  | soil P^f^ | Total organic soil phosphorus per dry mass | % |
|  | soil C:N^f^ | Organic soil C-to-N ratio | - |
|  | soil C:P^f^ | Organic soil C-to-P ratio | - |
|  | soil N:P^f^ | Organic soil N-to-P ratio | - |
|  | NH_4_-N^g^ | Soil inorganic N from ammonium | μg cm^-2^ year^-1^ |
|  | PO_4_-P^g^ | Soil inorganic P from phosphate | μg cm^-2^ year^-1^ |
|  | NH_4_-N: PO_4_-P^f^ | Soil ammonium to phosphate | - |

**The labeled trait data were obtained from the following online publications on SPRUCE site:**

**^a^**Warren, J. M. *et al.* Divergent species‐specific impacts of whole ecosystem warming and elevated CO_2_ on vegetation water relations in an ombrotrophic peatland. *Global change biology* 27, 1820-1835 (2021). **^b^**Richardson, A. *et al.* SPRUCE Vegetation Phenology in Experimental Plots from Phenocam Imagery, 2015-2022. (Oak Ridge National Laboratory (ORNL), Oak Ridge, TN (United States), 2023).**^c^**Dusenge, M. E. *et al.* Boreal conifers maintain carbon uptake with warming despite failure to track optimal temperatures. *Nature communications* 14, 4667 (2023). **^d^**Dusenge, M. E. *et al.* Warming induces divergent stomatal dynamics in co‐occurring boreal trees. *Global change biology* 27, 3079-3094 (2021). **^e^**Phillips, J., Hanson, P. & Warren, J. SPRUCE Plant Tissue Analyses Beginning 2017. (Oak Ridge National Laboratory, TES SFA, U.S. Department of Energy, Oak Ridge, Tennessee, U.S.A, 2021).**^f^**Kolka, R., Griffiths, N., Oleheiser, K. & Schadt, C. SPRUCE Mass and Chemistry of Peat Ladder Decomposition Treatments, 2017-2020. (Oak Ridge National Laboratory, TES SFA, U.S. Department of Energy, Oak Ridge, Tennessee, U.S.A, 2023).**^g^**Iversen, C. *et al.* SPRUCE plant-available nutrients assessed with ion-exchange resins in experimental plots, beginning in 2013. (Oak Ridge National Laboratory, TES SFA, U.S. Department of Energy, Oak Ridge, Tennessee, U.S.A, 2017).

**Table S2** Summary of the fitted generalized linear mixed models of BAI.

| **Regional-scale** | | | | | | **SPRUCE-experimental scale** | | | | | |
| --- | --- | --- | --- | --- | --- | --- | --- | --- | --- | --- | --- |
| **Fixed effects** | **Coef** | **LCI** | **UCI** | ***z-value*** | ***P-value*** | **Fixed effects** | **Coef** | **LCI** | **UCI** | ***z-value*** | ***P-value*** |
| (Intercepts) | **0.33** | 0.14 | 0.51 | 3.49 | <0.001 | (Intercepts) | -0.03 | -0.32 | 0.26 | -0.20 | 0.84 |
| BA | **0.52** | 0.47 | 0.57 | 19.57 | <0.001 | BA | **0.46** | 0.32 | 0.61 | 6.25 | <0.001 |
| spruce | **-0.27** | -0.35 | -0.19 | -6.23 | <0.001 | spruce | 0.01 | -0.24 | 0.27 | 0.10 | 0.92 |
| VPD | **-0.03** | -0.04 | -0.02 | -4.41 | <0.001 | VPD | **0.47** | 0.25 | 0.69 | 4.25 | <0.001 |
| T_soil_ | 0.01 | -0.01 | 0.03 | 1.12 | 0.26 | WT | 0.03 | -0.06 | 0.12 | 0.68 | 0.49 |
| SM | **0.16** | 0.11 | 0.21 | 6.67 | <0.001 | eCO_2_ | -0.19 | -0.65 | 0.27 | -0.81 | 0.42 |
| X1 | **0.13** | 0.05 | 0.20 | 3.22 | 0.001 | X1 | **-2.16** | -3.87 | -0.46 | -2.49 | 0.01 |
| X2 | **0.19** | 0.12 | 0.25 | 5.86 | <0.001 | X2 | -0.44 | -1.78 | 0.90 | -0.64 | 0.52 |
| X3 | **0.10** | 0.04 | 0.16 | 3.07 | 0.00 | X3 | **-1.97** | -3.57 | -0.38 | -2.42 | 0.02 |
| X4 | **0.20** | 0.14 | 0.26 | 6.57 | <0.001 | X4 | **3.26** | 0.91 | 5.61 | 2.72 | 0.007 |
| BA*spruce | **-0.10** | -0.16 | -0.03 | -2.91 | 0.004 | BA*spruce | -0.03 | -0.20 | 0.14 | -0.34 | 0.73 |
| VPD*spruce | **-0.02** | -0.03 | -0.01 | -3.40 | <0.001 | VPD*spruce | -0.41 | -0.62 | -0.21 | -4.04 | <0.001 |
| Tsoil*spruce | -0.01 | -0.03 | 0.01 | -0.92 | 0.36 | WT*spruce | 0.03 | -0.07 | 0.13 | 0.65 | 0.52 |
| SM*spruce | **-0.05** | -0.09 | -0.01 | -2.57 | 0.01 | eCO_2_*spruce | **0.50** | 0.08 | 0.92 | 2.32 | 0.02 |
| **Random effects** | | | | | | **Random effects** | | | | | |
| Grouping factor | | Random term | | | SD | Grouping factor |  | Random term | |  | SD |
| Site | | (Intercept) | | | 0.14 | Plot |  | TreeID |  |  | 0.41 |
| Site | | VPD | | | 0.05 | Plot |  | Plot |  |  | 0.22 |
| Site | | Tsoil | | | 0.04 |  |  |  |  |  |  |
| Site | | SM | | | 0.17 |  |  |  |  |  |  |
| TreeID | | (Intercept) | | | 0.47 |  |  |  |  |  |  |
| TreeID | | AR(1) ρ | | | 0.89 |  |  |  |  |  |  |
| TreeID | | AR(1) innovation SD | | | 0.60 |  |  |  |  |  |  |
| Spatial | | spatial effect (exp basis) | | | 0.75 |  |  |  |  |  |  |
| **Dispersion model** | **coef** | **LCI** | **UCI** | **z-value** | **P-value** | **Dispersion model** | **coef** | **LCI** | **UCI** | **z-value** | **P-value** |
| (Intercepts) | **-1.80** | -1.84 | -1.77 | -95.11 | <0.001 | (Intercepts) | **-0.24** | -0.24 | -0.23 | -126.63 | <0.001 |
| BA | **0.38** | 0.35 | 0.41 | 27.04 | <0.001 | BA | **0.46** | 0.45 | 0.46 | 244.78 | <0.001 |
| R^2^m/R^2^c | 0.30/0.99 | | | | | R^2^m/R^2^c 0.23/0.57 | | | | | |

Note: The fixed part of the model included species (larch vs. spruce), tree size (basal area; BA), vapor pressure deficit (VPD), soil temperature (T_soil_), soil moisture (SM), water-table depth (WT), CO_2_ treatment (eCO_2_ vs. aCO_2_), and interactions between species and the other fixed effects. The random part of the model included hierarchical grouping terms for site/plot and individual trees, with nesting applied where appropriate. The intercept corresponds to the reference species (larch) and ambient CO₂ (aCO₂). Terms x1–x4 represent coefficients of a cubic regression-spline basis for year (basis dimension 𝑘=10). At the regional scale, the response variable is log-transformed basal area increment (BAI) for 1970–2010, assuming a Gaussian distribution. Temporal autocorrelation within trees was modeled with an AR(1) structure; we report the AR(1) correlation parameter (𝜌) and the AR(1) innovation standard deviation (the stddev of the AR(1) block). Spatial dependence was modeled using an exponential spatial correlation structure (exp(pos + 0 | group)), where pos encodes unique longitude-latitude coordinate pairs and group defines a single spatial field. At the SPRUCE experiment, the response variable is BAI for 2017–2021, and a Tweedie distribution was used to accommodate zero values. R^2^_m_ and R^2^_c_ denote marginal (fixed effects) and conditional (fixed + random effects) R^2^, respectively. Coefs are standardized regression coefficients; LCI and UCI are 95% confidence intervals. z values are Wald statistics (Estimate/SE), with two-sided *P* values based on the standard normal distribution.

**Table S3** R^2^ explained by bivariate relationships between BAI, VPD, T_soil_, WT and nutrients. The BAI group denotes the impacts of soil and plant nutrients on BAI. VPD, T_soil,_ and soil water table depth (WT) groups denote their relationships with soil and plant nutrients. When n=4-5, permutation-based *P-values* were computed using an exact two-sided permutation test (all n! permutations). The plus sign in green (+) and minus sign in grey (−) denote positive and negative relationships, respectively. **+** or **-**, *P*<0.05; **+ +** or **- -**, *P*<0.01; **+ + +** or **- - -**, *P*<0.001. The plus or minus signs in blue mean *P*=0.06.

|  |  | **Organic soil nutrients** | | | | | | **Inorganic soil nutrients** | | | **Plant nutrients** | | | | |
| --- | --- | --- | --- | --- | --- | --- | --- | --- | --- | --- | --- | --- | --- | --- | --- |
|  |  | **soil C** | **soil N** | **soil P** | **soil C:N** | **soil C:P** | **soil N:P** | **NH_4_-N** | **PO_4_-P** | **NH_4_-N:PO_4_-P** | **N_leaf.A_** | **leaf P** | **leaf C:P** | **stem C:P** | **leaf N:P** |
| **eCO_2_ spruce** | **BAI** | **R^2^=0.85, -** |  | **R^2^=0.92, +** |  | **R^2^=0.88, --** | **R^2^=0.86, -** |  |  | **R^2^=0.83, +** |  |  |  |  |  |
| **aCO_2_ spruce** | **BAI** |  |  |  |  |  |  |  |  |  |  |  |  |  |  |
| **eCO_2_ larch** | **BAI** |  |  |  |  |  |  |  |  |  | **R^2^=0.92, +** | **R^2^=0.47, -** | **R^2^=0.67, + +** |  | **R^2^=0.72, + +** |
| **aCO_2_ larch** | **BAI** |  |  |  |  |  |  |  |  |  |  | **R^2^=0.51, +** | **R^2^=0.48, -** |  |  |
| **all spruce** | **BAI** | **R^2^=0.60, - -** |  | **R^2^=0.50, +** |  | **R^2^=0.54, -** | **R^2^=0.58, -** |  |  | **R^2^=0.49, +** |  |  |  |  |  |
| **all larch** | **BAI** |  | **R^2^=0.51, +** |  | **R^2^=0.60, - -** |  |  |  |  | **R^2^=0.43, +** |  |  |  |  |  |
| **eCO_2_** | **VPD** |  |  |  |  |  |  |  |  | **R^2^=0.83, +** | **L: R^2^=0.85, +** |  | **L: R^2^=0.43, +** |  | **L: R^2^=0.42, +** |
| **aCO_2_** | **VPD** |  |  |  |  |  |  | **R^2^=0.81, +** | **R^2^=0.93, + +** |  |  | **S: R^2^=0.61, ++** | **S: R^2^=0.55, -** |  |  |
| **all CO_2_** | **VPD** |  |  |  | **R^2^=0.42, -** |  |  | **R^2^=0.63, + +** |  |  |  | **S: R^2^=0.25, +** | **S: R^2^=0.21, -** | **S: R^2^=0.20, -** |  |
| **eCO_2_** | **T_soil_** |  |  |  |  |  |  |  |  | **R^2^=0.83, +** | **L: R^2^=0.74, +** |  |  |  |  |
| **aCO_2_** | **T_soil_** |  |  |  |  |  |  | **R^2^=0.87, +** | **R^2^=0.96, + +** |  |  | **S: R^2^=0.54, +** | **S: R^2^=0.47, -** |  |  |
| **all CO_2_** | **T_soil_** |  |  |  |  |  |  | **R^2^=0.74, + +** | **R^2^=0.40, +** |  |  | **S: R2=0.25, +** | **S: R^2^=0.21, -** |  |  |
| **eCO_2_** | **WT** |  |  |  |  |  |  |  |  | **R^2^=0.94, -** |  | **L: R^2^=0.66, ++** | **L: R^2^=0.71, --** |  | **L: R^2^=0.72, --** |
| **aCO_2_** | **WT** |  |  |  |  |  |  |  |  |  |  | **S: R^2^=0.63, --** | **S:R^2^=0.53,+** |  | **S:R^2^=0.45,+** |
| **all CO_2_** | **WT** |  |  |  |  |  |  |  |  | **R^2^=0.49,-** |  |  |  | **S: R^2^=0.18, +** |  |

Note: L and S indicate larch and spruce, respectively. Leaf N refers to leaf nitrogen content and N_leaf.A_ refers to leaf-area-based nitrogen content.

**Table S4** R^2^ explained by bivariate significant relationships between soil nutrients and plant nutrients. The plus sign in green (+) and minus sign in grey (−) denote positive and negative relationships, respectively. **+** or **-**, *P*<0.05; **+ +** or **- -**, *P*<0.01; **+ + +** or **- - -**, *P*<0.001. The plus or minus signs in blue mean *P*=0.06. R^2^ for nonsignificant relationships were not shown. For trait abbreviations, see Supplementary Table 1.

|  | | **N_leaf.A_** | **leaf N** | **stem N** | **leaf P** | **leaf C:N** | **stem C:N** | **leaf P** | **leaf N:P** | **stem N:P** | **leaf C:P** | **stem C:P** |
| --- | --- | --- | --- | --- | --- | --- | --- | --- | --- | --- | --- | --- |
| **aCO_2_ spruce** | **soil N** | **R^2^=0.81, +** |  |  |  |  |  |  |  |  | **R^2^=0.39, -** |  |
| **all spruce** | **soil N** | **R^2^=0.49, +** | **R^2^=0.38, ++** |  |  | **R^2^=0.35,--** |  |  |  |  |  |  |
| **all larch** | **soil N** |  |  | **R^2^=0.21, +** |  |  | **R^2^=0.20, -** |  |  | **R^2^=0.20, +** |  |  |
| **eCO_2_ spruce** | **NH_4_-N** |  |  |  | **R^2^=0.38, +** |  |  |  |  |  |  | **R^2^=0.39, -** |
| **aCO_2_ spruce** | **NH_4_-N** | **R^2^=0.75, +** |  |  | **R^2^=0.44, +** |  |  |  |  |  | **R^2^=0.42,-** |  |
| **all spruce** | **NH_4_-N** |  | **R^2^=0.19, +** |  | **R^2^=0.39, +** | **R^2^=0.18, +** |  |  |  |  | **R^2^=0.38,-** |  |
| **eCO_2_ larch** | **soil P** |  |  |  | **R^2^=0.53,-** |  |  |  | **R^2^=0.51, +** |  | **R^2^=0.50, +** |  |
| **aCO_2_ spruce** | **PO_4_-P** | **R^2^=0.82, +** |  |  |  |  |  | **R^2^=0.41, +** |  |  | **R^2^=0.42,-** |  |
| **all spruce** | **PO_4_-P** |  |  |  | **R^2^=0.31, +** |  |  |  |  |  | **R^2^=0.31,-** |  |
| **all larch** | **PO_4_-P** |  |  |  | **R^2^=0.23, +** |  |  |  |  |  |  |  |
| **eCO_2_ larch** | **soil C:P** |  |  |  | **R^2^=0.50, +** |  |  |  | **R^2^=0.46,-** |  | **R^2^=0.46,-** |  |
| **aCO_2_ larch** | **soil C:P** | **R^2^=0.91, +** |  |  |  |  |  |  |  |  |  |  |
| **eCO_2_ spruce** | **soil N:P** |  |  |  |  |  |  |  |  |  |  | **R^2^=0.39, +** |
| **aCO_2_ larch** | **soil N:P** | **R^2^=0.73, +** |  |  |  |  |  |  |  |  |  |  |
| **aCO_2_ spruce** | **soil C:N** | **R^2^=0.90, +** |  |  |  |  |  |  |  |  | **R^2^=0.37, +** |  |
| **eCO_2_ larch** | **soil C:N** |  |  |  |  |  |  |  | **R^2^=0.45,-** |  |  |  |
| **all spruce** | **soil C:N** | **R^2^=0.48, +** | **R^2^=0.34,--** |  |  | **R^2^=0.30, +** |  |  |  |  |  |  |
| **all larch** | **soil C:N** |  |  | **R^2^=0.20,-** |  |  | **R^2^=0.19, +** |  |  | **R^2^=0.20,-** |  |  |
| **eCO_2_ larch** | **NH_4_-N:PO_4_-P** |  |  |  | **R^2^=0.55,-** |  |  |  | **R^2^=0.60, +** |  | **R^2^=0.68, ++** |  |
| **all larch** | **NH_4_-N:PO_4_-P** | **R^2^=0.47, +** |  |  | **R^2^=0.36,--** |  |  |  | **R^2^=0.30, +** |  | **R^2^=0.52, +++** |  |

**Table S5** Summary of an analysis of covariance (ANCOVA), where summer VPD is a covariate, CO_2_ treatment and species as fixed factors, testing the relationship between trait values and summer VPD. The regression was selected based on the lowest adjusted Akaike information criterion (AICc) in Extended Data Table 8. Non-significant regressions were not shown.

| **Trait** | **Intercept** | **Slope (VPD)** | **eCO_2_** | **spruce** | **VPD:spruce** | **VPD:eCO_2_** | **Spruce:eCO_2_** | **VPD:eCO_2_:spruce** | ***R^2^*** | ***P*-value** |
| --- | --- | --- | --- | --- | --- | --- | --- | --- | --- | --- |
| BAI~VPD | -0.31 | 1.09** |  |  |  |  |  |  | 0.37 | 0.006 |
| N_leaf.A_~VPD+CO_2_+species | 0.95*** | 0.21* | -0.25* | 0.66*** |  |  |  |  | 0.75 | <0.001 |
| N_leaf_~VPD+CO_2_+species | 0.98*** | 0.15** | -0.29*** | -0.43*** |  |  |  |  | 0.84 | <0.001 |
| NH_4_-N:PO_4_-P~VPD+CO_2_ | 2.85** | -0.22 | -3.25* |  |  | 1.54* |  |  | 0.75 | 0.03 |
| N_stem.uptake_~VPD* species | -0.004* | 0.004*** |  |  | -0.004*** |  |  |  | 0.73 | <0.001 |
| K_s_~VPD+CO_2_*species | 3.06** | 1.02* | -2.40* | -4.13*** |  |  | 3.27* |  | 0.73 | <0.001 |
| E_t_~VPD*species | 4.07 | 8.21* |  | 0.11 | -6.13* |  |  |  | 0.84 | <0.001 |
| Ψ_50_~VPD*CO_2_*species | -3.67*** | 0.06 | 1.89** | -0.69 | 0.50 | -0.70* | -2.13* | 0.51 | 0.84 | 0.001 |
| Ψ_pre_~VPD*species | -0.29 | -0.68*** |  | 0.03 | 0.62** |  |  |  | 0.88 | <0.001 |
| HSM~VPD+CO_2_*species | 1.37*** | -0.15 | -0.37 | 0.47 |  |  | 1.14** |  | 0.75 | <0.001 |
| Ψ_pre_-Ψ_min_~VPD*species | 1.86*** | -0.54*** |  | -0.30 | 0.41* |  |  |  | 0.66 | <0.001 |
| PLFC~VPD*species | 0.63*** | 0.12* |  | 0.04 |  |  | -0.13 |  | 0.69 | <0.001 |
| GSL~VPD+CO_2_+species | 151.23*** | 18.93*** | -7.05* | 48.16*** |  |  |  |  | 0.97 | <0.001 |
| Green-up~VPD+species | 127.34*** | -9.23*** |  | -26.83*** |  |  |  |  | 0.96 | <0.001 |
| Green-down~VPD+CO_2_+species | 276.96*** | 9.60*** | -5.20* | 21.22*** |  |  |  |  | 0.91 | <0.001 |
| A_25_~VPD+CO_2_+species | 6.41*** | 1.44* | 2.12* | -2.41** |  |  |  |  | 0.57 | 0.003 |
| iWUE~VPD*CO_2*_species | 66.87*** | -5.27 | 42.22** | 41.54** | 1.94 | -14.05* | -12.36 | 25.52* | 0.98 | <0.001 |
| T_optV_~VPD+ species | 31.51*** | 1.87*** |  | -1.46* |  |  |  |  | 0.63 | <0.001 |
| g_s_~VPD+ species | 0.12*** | 0.03** |  | -0.11*** |  |  |  |  | 0.84 | <0.001 |
| V_cmax_~VPD+CO_2_+species | 41.24*** | 3.86 | -14.77*** | -0.19 |  |  |  |  | 0.57 | 0.003 |
| SLA~VPD+CO_2_+species | 227.66*** | -3.91 | -21.67 | -127.34*** |  |  |  |  | 0.84 | <0.001 |
| LDMC~VPD+CO_2_+species | 0.40*** | -0.01 | 0.02 | 0.07*** |  |  |  |  | 0.66 | <0.001 |
| Aba.SD~VPD+CO_2_+species | 50.56* | 8.73 | 8.12 | -39.47** |  |  |  |  | 0.52 | 0.01 |
| Ada.SD~VPD+CO_2_+species | 30.23* | 0.62 | -0.93 | 61.82*** |  |  |  |  | 0.82 | <0.001 |
| Aba.guard~VPD*CO_2_+species | 28.85*** | -3.46* | -9.95* | 8.03*** |  | 6.75* |  |  | 0.74 | <0.001 |
| Aba.guard~VPD+CO_2_+species | 23.27*** | 0.59 | 1.96 | 6.85** |  |  |  |  | 0.48 | 0.03 |
| Ada.SPI~VPD+CO_2_+species | -4.15*** | 0.10 | 0.20 | 1.62*** |  |  |  |  | 0.87 | <0.001 |
| SPI~VPD+CO_2_+species | -3.42*** | 0.25 | 0.23 | 1.08*** |  |  |  |  | 0.61 | 0.002 |
| WD~VPD+CO_2_+species | 0.51*** | -0.02 | 0.001 | 0.06* |  |  |  |  |  |  |
| Tw.l | 3.34* | 0.11 | -0.77 | -0.33 |  |  | 1.30* |  |  |  |
| DPA~VPD+CO_2_+species | 4.82*** | 0.02 | 0.17 | -0.77** |  |  |  |  | 0.42 | 0.04 |
| DT~VPD+CO_2_+species | 7.98*** | 0.14 | 0.02 | -1.39*** |  |  |  |  | 0.62 | 0.002 |
| DPM~VPD+CO_2_+species | 13.27*** | 0.81 | 0.10 | -2.55*** |  |  |  |  | 0.62 | 0.002 |
| MF~VPD | 0.40*** | 0.02** |  |  |  |  |  |  | 0.35 | 0.007 |

**Table S6** Model comparison between models with interaction of the main treatment effects and models with only main treatment effects using adjusted Akaike information criterion (AICc). For trait abbreviations, see Supplementary Table 1.

| Abbreviation | VPD*CO_2_*Species | VPD+CO_2_+Species | VPD*CO_2_+Species | VPD+CO_2_*Species | VPD*Species+CO_2_ | VPD*Species+CO_2_*Species |
| --- | --- | --- | --- | --- | --- | --- |
| BAI | 86.62 | **69.18** | 70.03 | 71.89 | 71.71 | 72.95 |
| N_leaf.A_ | 29.93 | **10.77** | 13.79 | 14.79 | 14.89 | 18.60 |
| N | 4.76 | **-13.62** | -9.81 | -12.21 | -9.57 | -5.06 |
| N_stem.uptake_ | -176.15 | -178.92 | -175.37 | -175.09 | **-190.39** | -186.91 |
| K_s_ | 77.04 | 79.38 | 83.72 | **74.96** | 77.14 | 82.31 |
| E_t_ | 152.42 | 141.17 | 142.03 | 144.87 | **139.65** | 143.28 |
| Ψ_50_ | **27.23** | 34.76 | 38.43 | 29.76 | 34.89 | 38.12 |
| Ψ_pre_ | 34.35 | 24.51 | 28.34 | 28.69 | 17.74 | 22.01 |
| Ψ_min_ | 15.38 | **3.06** | 7.15 | 7.21 | 4.21 | 8.98 |
| HSM | 40.41 | 38.17 | 41.23 | **33.02** | 40.53 | 43.73 |
| Ψ_pre_-Ψ_min_ | 25.93 | 17.26 | 21.19 | 21.43 | **15.26** | 19.80 |
| PLFC | -6.49 | -24.42 | -20.22 | -20.27 | **-24.74** | -19.60 |
| Growing season | 148.52 | **128.58** | 130.85 | 132.28 | 132.51 | 135.74 |
| Green-up | 124.09 | **105.95** | 110.30 | 108.93 | 107.57 | 112.75 |
| Green-down | 132.45 | **118.70** | 119.96 | 119.13 | 123.01 | 124.87 |
| A_25_ | 108.00 | **89.90** | 92.26 | 94.01 | 92.83 | 95.77 |
| iWUE | **158.43** | 170.11 | 174.26 | 160.08 | 170.37 | 175.21 |
| T_optV_ | 96.01 | **74.75** | 78.89 | 78.91 | 78.81 | 83.65 |
| g_s_ | -58.72 | **-75.91** | -72.71 | -71.74 | -74.84 | -71.12 |
| V_cmax_ | 166.09 | **147.76** | 149.38 | 151.44 | 151.83 | 154.14 |
| SLA | 213.51 | 195.42 | 199.80 | 199.35 | **194.84** | **199.99** |
| LDMC | -48.61 | **-69.19** | -65.31 | -65.04 | -66.42 | -62.05 |
| Aba.SD | 204.54 | **183.29** | 187.64 | 187.67 | 186.36 | 191.42 |
| Ada.SD | 172.95 | **137.17** | 142.83 | 141.67 | 142.91 | 150.21 |
| Aba.Epidermal | 225.79 | **205.94** | 209.08 | 210.03 | 210.20 | 214.20 |
| Ada.Epidermal | 205.92 | 192.34 | 193.87 | 196.49 | **190.34** | 192.72 |
| Aba.Guard | 126.09 | 116.19 | **111.58** | 120.55 | 120.57 | 116.64 |
| Ada.Guard | 130.61 | **108.77** | 113.55 | 113.58 | 110.17 | 116.15 |
| Aba.SPI | 54.08 | **33.77** | 36.83 | 37.99 | 37.60 | 45.65 |
| Ada.SPI | 54.24 | **23.52** | 29.12 | 27.14 | 27.84 | 44.86 |
| SPI | 60.92 | **38.28** | 42.37 | 42.48 | 42.63 | 51.72 |
| WD | -31.95 | **-46.33** | -44.38 | -42.76 | -43.49 | -41.59 |
| Dh.e | 100.62 | **81.96** | 86.22 | 86.34 | 86.34 | 91.40 |
| Dh.l | 112.68 | **94.62** | 96.87 | **98.27** | 98.82 | 101.98 |
| TD.e | 339.99 | **326.09** | 326.11 | 328.78 | 330.44 | 331.06 |
| TD.l | 368.23 | **350.95** | 351.24 | 354.63 | 355.30 | 356.20 |
| LF.e | 8.31 | **-12.85** | -10.04 | -8.99 | -8.59 | -5.12 |
| LF.l | -6.57 | -26.68 | -22.63 | -22.66 | -22.63 | -17.87 |
| Tw.e | 61.24 | **39.83** | 43.81 | 42.55 | 44.22 | 48.98 |
| Tw.l | 55.54 | 48.32 | 52.52 | **45.87** | 52.63 | 57.66 |
| DPA | 56.68 | **38.69** | 42.62 | 41.67 | 42.45 | 47.29 |
| DT | 64.33 | **45.76** | 50.09 | 50.11 | 47.62 | 52.80 |
| DPM | 91.43 | **72.14** | 76.51 | 75.68 | 75.35 | 80.47 |
| MF | -63.33 | **-80.68** | -76.67 | -80.53 | -77.63 | -72.70 |

**Fig. S1** Effects of climate variables on basal area growth for larch and spruce at the SPRUCE Ecosystem experiment from 2017 to 2021. **a,** Growth-climate relationships for air temperature (T_air_), soil water table depth from surface (WT), and elevated CO_2_ (eCO_2_). **b**, Growth-climate relationships for T_soil_, WT and eCO_2_. Tree size indicates basal area. The x axes indicate the values of standardized coefficients with error bars (95% confidence intervals), and the y axes indicate climate and tree size factors. Dashed lines indicate that the standardized coefficients are not different from zero.

**Fig. S2** Significant relationships between BAI and physiological traits. Red and teal indicate larch and spruce, respectively. Filled and open symbols indicate elevated (eCO_2_) and ambient CO_2_ (aCO_2_), respectively. Solid, long-dashed, and dotted lines indicate significant regressions across CO₂ treatments, under aCO₂, and under eCO₂, respectively. Bold, italic, and regular R^2^ values correspond to regressions across CO_2_ treatments, under aCO_2_, and under eCO_2_, respectively. When n=4-5, permutation-based *P*-values are shown; they were computed using an exact two-sided permutation test (all n! permutations), defined as the fraction of permutations with an absolute slope at least as large as the observed value. All regressions are based on linear regressions, and data were log-transformed when needed (non-linear lines) to improve normality and homoscedasticity. *, *P*<0.05; **, *P*<0.01; ***, *P*<0.001. For trait abbreviations, see Table S1.

**Fig. S3** Climate-driven soil nutrients affect tree growth. **a-f**, climate variables including VPD, T_soil,_ and soil water table depth affect soil and plant nutrients. Black symbols indicate eCO_2_ and grey symbols indicate aCO_2_. Black, grey, and red lines indicate the regression for eCO_2_, aCO_2_, and all CO_2_ treatments, respectively. **g-k**, soil and leaf nutrients impact tree growth; red and teal indicate larch and spruce, respectively. Filled and open symbols indicate elevated (eCO_2_) and ambient CO_2_ (aCO_2_), respectively. Solid, long-dashed, and dotted lines indicate significant regressions across CO₂ treatments, under aCO₂, and under eCO₂, respectively. Bold, italic, and regular R^2^ values correspond to regressions across CO_2_ treatments, under aCO_2_, and under eCO_2_, respectively. NH_4_:PO_4_, soil inorganic nitrogen to phosphorus ratio; soil C:N, soil organic carbon to nitrogen ratio; soil C:P, soil organic carbon to phosphorus ratio. When n=4-5, permutation-based *P-values* are shown; they were computed using an exact two-sided permutation test (all n! permutations). All regressions are based on linear regressions, and data were log-transformed when needed (non-linear lines) to improve normality and homoscedasticity. *, *P*<0.05; **, *P*<0.01; ***, *P*<0.001.

**Fig. S4** Heatmap showing pairwise Pearson correlation coefficients (r) among 35 traits within larch (n=9). Cell color indicates the magnitude and direction of r. Warm (red) shades indicate positive correlations and cool (blue) shades indicate negative correlations. Rows and columns were hierarchically clustered (complete linkage) using 1−|r| as the distance metric. For trait abbreviations, see Table S1.

**Fig. S5** Significant relationships between relative growth rate (RGR) and physiological traits. Red and teal indicate larch and spruce, respectively. Filled and open symbols indicate elevated (eCO_2_) and ambient CO_2_ (aCO_2_), respectively. Solid, long-dashed, and dotted lines indicate significant regressions across CO₂ treatments, under aCO₂, and under eCO₂, respectively. Bold, italic, and regular R^2^ values correspond to regressions across CO_2_ treatments, under aCO_2_, and under eCO_2_, respectively. When n=4-5, permutation-based *P*-values are shown; they were computed using an exact two-sided permutation test (all n! permutations), defined as the fraction of permutations with an absolute slope at least as large as the observed value. All regressions are based on linear regressions, and data were log-transformed when needed (non-linear lines) to improve normality and homoscedasticity. *, *P*<0.05; **, *P*<0.01; ***, *P*<0.001. For trait abbreviations, see Table S1.

**Fig. S6** Wood anatomical traits determine hydraulics. **a-d**, hydraulic efficiency. **e-h**, hydraulic safety. Red and teal indicate larch and spruce, respectively. Filled and open symbols indicate elevated (eCO_2_) and ambient CO_2_ (aCO_2_), respectively. Solid, long-dashed, and dotted lines indicate significant regressions across CO₂ treatments, under aCO₂, and under eCO₂, respectively. Bold, italic, and regular R^2^ values correspond to regressions across CO_2_ treatments, under aCO_2_, and under eCO_2_, respectively. When n=4, permutation-based *P-values* are shown; they were computed using an exact two-sided permutation test (all n! permutations). All regressions are based on linear regressions, and data were log-transformed when needed (non-linear lines) to improve normality and homoscedasticity. R^2^ for each regression line is shown. *, *P*<0.05; **, *P*<0.01; ***, *P*<0.001. For trait abbreviations, see Table S1.

**Fig. S7** Heatmap showing pairwise Pearson correlation coefficients (r) among 35 traits within spruce (n=10). Cell color indicates the magnitude and direction of r. Warm (red) shades indicate positive correlations and cool (blue) shades indicate negative correlations. Rows and columns were hierarchically clustered (complete linkage) using 1−|r| as the distance metric. *, 0.01<*P*<0.05; **, 0.001<*P*<0.01; ***, *P*<0.001. For trait abbreviations, see Table S1.

**Fig. S8** Impacts of vapor pressure deficit (VPD) and CO_2_ on physiological traits related to hydraulics and carbon assimilation. Red and teal indicate larch and spruce, respectively. Filled and open symbols indicate elevated (eCO_2_) and ambient CO_2_ (aCO_2_), respectively. Solid, long-dashed, and dotted lines indicate significant regressions across CO₂ treatments, under aCO₂, and under eCO₂, respectively. Bold, italic, and regular R^2^ values correspond to regressions across CO_2_ treatments, under aCO_2_, and under eCO_2_, respectively. Equations for the best-fitting model, based on AICc and R^2^, are shown. Significant differences in interceptions are indicated by separate regression lines within each trait. For full statistical results, see Table S5. *, *P*<0.05; **, *P*<0.01; ***, *P*<0.001. For trait abbreviations, see Table S1.

**Fig. S9** Impacts of VPD on trait differences between eCO_2_ and aCO_2_ (Δ, see equation in panel a). **a-b**, significant impacts of VPD on Δ for larch. **c-f**, impacts of VPD on Δ for spruce. Red and teal indicate larch and spruce, respectively. Filled and open symbols indicate elevated (eCO_2_) and ambient CO_2_ (aCO_2_), respectively. Solid, long-dashed, and dotted lines indicate significant regressions across CO₂ treatments, under aCO₂, and under eCO₂, respectively. Due to the limited number of trees available to sample in some experimental treatments, only four plots were sampled for certain parameters of larch under eCO_2_ (dashed lines). All regressions are linear, and data were log-transformed when needed (nonlinear lines) to improve normality and homoscedasticity. R^2^ for each regression line is shown. *, *P*<0.05; **, *P*<0.01; ***, *P*<0.001.
